# Origins of Graphite Resistivity: Decoupling Stacking Fault and Rotational Misorientation

**DOI:** 10.1002/advs.202518254

**Published:** 2026-01-07

**Authors:** Weipeng Chen, Fuwei Yang, Tielin Wu, Yelingyi Wang, Quanshui Zheng, Deli Peng, Zhanghui Wu

**Affiliations:** ^1^ College of Mechatronics and Control Engineering Shenzhen University Shenzhen China; ^2^ Center for Nano and Micro Mechanics Tsinghua University Beijing China; ^3^ Department of Engineering Mechanics School of Aerospace Engineering Tsinghua University Beijing China; ^4^ Institute of Superlubricity Technology Research Institute of Tsinghua University in Shenzhen Shenzhen China; ^5^ Tsinghua Shenzhen International Graduate School Shenzhen China

**Keywords:** c‐axis transport, electrical resistivity, graphite, rotational misorientations, stacking faults, van der Waals interfaces

## Abstract

Van der Waals (vdW) layered materials, distinguished by weak interlayer interactions and diverse electronic properties, have ignited widespread research in materials science, physics, and device engineering. Interfacial dislocations, such as stacking faults (SF) and rotational misorientations (RM), are typically unavoidable and play a key role in shaping their interlayer transport properties. However, the absence of techniques to decouple their individual contributions has hindered a quantitative understanding of how stacking states affect interlayer electrical transport, even in well‐studied materials like graphite. In this work, we measure the intrinsic c‐axis resistivity of AB‐stacked epitaxial single‐crystal graphite (5.7 × 10^−5^ Ω · m) through high‐throughput measurements at room temperature. We further develop a decoupling strategy that combines rotational locking of highly oriented pyrolytic graphite (HOPG) with in situ measurements. Comparative analysis enable us to quantify, for the first time, the interlayer effective resistivity ratio of RM:SF:AB stacking as approximately 4507:74:1. In addition, we extend our methodology by introducing a large‐scale, pixel‐array‐based lateral measurement technique that reveals the internal dislocation structures and their tunability in vdW materials from a new spatial perspective. This work marks a significant advance in elucidating graphite's c‐axis electrical transport, providing a robust framework for investigating vdW materials with complex stacking structures.

## Graphical abstract

Interfacial dislocations critically influence interlayer transport in van der Waals (vdW) materials, yet quantifying their individual contributions remains challenging. We measure graphite's c‐axis resistivity and develop a decoupling strategy, revealing a resistivity ratio of ∼4507:74:1 for rotational misorientations, stacking faults, and AB stacking, which presents a robust methodology for deciphering interlayer phenomena in vdW materials featuring intricate stacking structures.

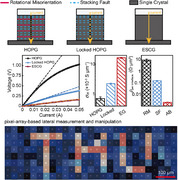

## 1 Introduction

Van der Waals (vdW) layered materials, such as graphite, have garnered widespread attention across physics, materials science, and device engineering due to their weak interlayer interactions and diverse electronic properties [1, 2, 3, 4]. These interactions enable interfacial reconfigurability, giving rise to a host of emergent phenomena, including anomalous superconductivity in magic‐angle graphene superlattices [5], structural superlubricity (SSL) in incommensurate vdW interfaces [6, 7, 8], angle‐dependent interfacial electrical/thermal transport [9, 10, 11, 12, 13], and enhanced fracture toughness in twisted configurations [14]. Interfacial dislocations in these materials are broadly classified as stacking faults (SF) and rotational misorientations (RM) [15, 16]. These interfacial features reshape the band structure and interlayer coupling, profoundly affecting out‐of‐plane charge transport. Notably, certain stacking configurations, such as rhombohedral graphite, are linked to exotic states including superconductivity and the quantum anomalous Hall effect. Understanding their role is particularly crucial for the design of vertical vdW devices, where c‐axis conduction is central. However, a systematic understanding of their respective impacts on electrical transport remains elusive—particularly along the c‐axis, where current passes through multiple interfaces. Despite nearly a century of investigation, graphite—the structurally simplest and most ubiquitous vdW layered material—lacks a comprehensive model of its c‐axis electrical transport, owing to two persistent challenges:

(1) Absence of a consistent intrinsic c‐axis resistivity benchmark: The intrinsic c‐axis resistivity, crucial for theoretical modeling and materials design, remains poorly defined. Room‐temperature measurements vary widely: 0.01–0.03 Ω∙m for natural graphite (NG) from Ceylon [17, 18], 3.0–6.7 × 10^−^^5^ Ω∙m for NG from Ticonderoga [19, 20, 21], 3.8–4.4 × 10^−^^5^ Ω∙m for NG from Gouveneur Talc [22], 6.5–7.6 × 10^−^^5^ Ω∙m for kish graphite (KG) [23], and 4–33 × 10^−4^ Ω∙m for natural graphite sheets [24]. Even NG, traditionally viewed as a high‐quality single crystal, contains internal defects—such as holes, claws, dislocations, SF, RM, and Newton‐ring‐like imperfections [25, 26, 27] that obscure these values [28, 29]. Theoretical estimates for ideal AB‐stacked graphite further diverge, yielding 4.8 × 10^−^^5^ Ω∙m [30] and 8.4 × 10^−^^5^ Ω∙m [31], depending on the band structure parameters employed, underscoring the need for a reliable standard.

(2) Lack of decoupling between SF and RM effects: Early studies of graphite's c‐axial electrical transport treated SF as uniformly distributed intercalations [31, 32, 33], largely neglecting the influence of RM. Only recently has RM's influence gained attention, with research shifting toward angle‐dependent studies of individual rotational dislocation interfaces [9, 10, 34, 35, 36, 37]. This shift reveals that prior SF measurements likely conflated RM contributions, necessitating a revised, systematic approach to decouple their roles.

Recent advances in materials science and micro/nanoscale technology offer powerful tools to address these challenges: epitaxially grown single‐crystal graphite (ESCG) offers a near‐ideal experimental benchmark [38]; micro‐fabricated pillar‐patterning techniques enable high‐throughput sample preparation [39, 40]; a recently developed microtip‐based four‐wire method allows precise electrical measurement and rotational control for micro pillars [41]. Our results demonstrate that the intrinsic resistivity of graphite with AB stacking is approximately 5.7 × 10^−5^ Ω · m at room temperature, and interior transport dominates when the characteristic size exceeds 2 µm. Furthermore, by conducting electrical measurements on highly oriented pyrolytic graphite (HOPG) pillars with rotational locking, we have revealed that RM account for approximately 68% of the graphite resistance, while SF contribute the remaining 27%. Strikingly, the effective interfacial resistance ratio among RM, SF, and AB stacking configurations is 4507:74:1. Additionally, our large‐scale lateral measurements further supplement the lateral homogeneity of ESCG and the tunability of the incommensurate interfaces inside HOPG.

## 2 Results

**FIGURE 1:**
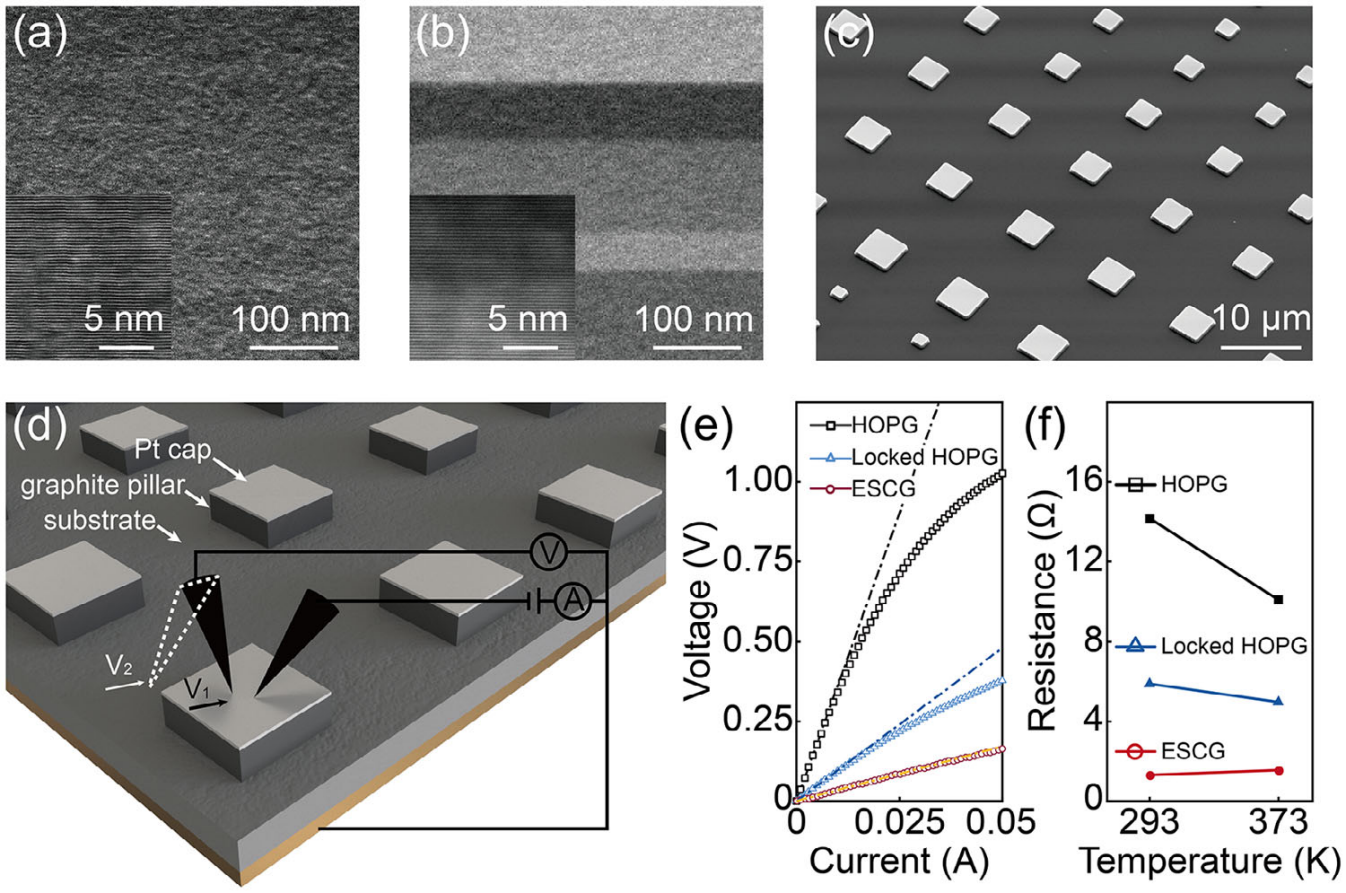
Overview of sample preparation and measurement for c‐axis electrical transport in graphite. (a,b) Cross‐sectional STEM images of ESCG with uniform stacking state (a) and HOPG with chaotically twisted grain boundaries (b) correspondingly. (c) Fabricated graphite micropillar arrays with Pt cap. (d) Schematic of the four‐wire resistance measurement based on two micro‐tips. (e) Representative *V/I* curves for ESCG, HOPG, and rotationally locked HOPG samples. (f) Temperature‐dependent resistance profiles for ESCG, HOPG, and locked HOPG, highlighting their distinct transport behaviors.

To establish a comprehensive understanding of c‐axis electrical transport in graphite, we investigated two representative graphite materials with distinct structural characteristics. (1) ESCG, was synthesized via isothermal carbon diffusion on single‐crystal nickel (Ni) substrates, enabling continuous epitaxial growth [38]. This material exhibits uniform AB stacking along the thickness direction, representing the highest‐quality single‐crystal graphite currently available (Figure 1a). (2) HOPG closely resembles single‐crystal graphite in its properties but possesses a fundamentally different stacking structure, characterized by interface dislocation with random spacings of several tens of nanometers that disrupt the normal stacking sequence (Figure 1b). Subsequently, we fabricated arrays of graphite micropillars (Figure 1c), each capped with a 100 nm‐thick Pt layer (see Methods and Figure S1). The Pt cap serves a dual purpose: it enhances the normal stiffness to prevent deformation during manipulation and ensures stable electrical contact between the graphite and the probe during measurements. The micropillars were precisely controlled in dimension, with graphite layer thicknesses ranging from 100–900 nm and lateral sizes between 4 and 10 µm. This approach not only facilitated high‐throughput sample preparation and testing, but also provided an ideal platform for investigating transport properties with microscale lateral size. Finally, electrical measurements (Figure 1d) were performed using four‐wire resistance measurement with two tungsten micro‐tips (detailed in Methods and Figure S2), which fundamentally eliminates interference from contact and substrate resistance, ensuring high accuracy and reliability in the experimental data at microscale. Notably, our measurements were conducted on thick graphite pillars at charge neutral point [42], in the absence of external gating or chemical doping.

The c‐axis electrical transport properties were determined through analysis of the measured voltage–current (*V*/*I*) characteristics, with three representative curves shown in Figure 1e. ESCG samples exhibit excellent Ohmic resistance behavior, indicating a linear relationship between current and voltage. In contrast, both standard HOPG and rotationally locked HOPG samples (discussed in detail below) display varying degrees of nonlinearity, which we attribute to the influence of SF and RM. For consistency in our analysis, all subsequent resistance values were derived from the voltage–current ratio at 5 mA. No obvious pressure dependence was observed in either ESCG or HOPG samples, as verified in Figure S3. The influence of contact pad size and position is detailed discussed in Discussion S1.

It is important to note that the structural dependence of c‐axis resistivity discussed herein is established within the context of phonon contributions at room temperature. This is highlighted by the temperature‐dependent behaviors shown in Figure 1f. The HOPG and locked HOPG samples display a negative temperature correlation, indicating that transport is limited by interfacial energy barriers (RMs and SFs); here, phonon‐assisted hopping or tunneling at higher temperatures facilitates carrier transport across these barriers, leading to reduced resistance. Conversely, ESCG displays a positive temperature correlation characteristic of metallic‐like coherent transport, where phonon scattering disrupts the coherent electron paths in the perfect AB stacking (see Discussion S2 for details). Furthermore, complementary low‐temperature measurements on bulk samples, combined with historical studies, reinforce that phonon contributions exerts a significant—yet diametrically opposed—influence on the c‐axis transport of HOPG vs. ESCG over a wide temperature range (see Discussion S3).

**FIGURE 2:**
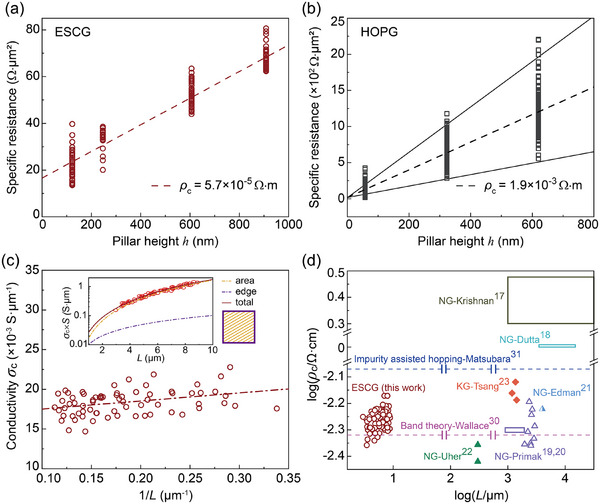
The intrinsic c‐axis electrical resistivity of graphite. (a,b) Specific resistance *r*_t_ = *R∗S* vs. pillars height *h* (not including Pt cap) for ESCG and HOPG, respectively. Each data point represents a single measurement on an independent pillar. The solid lines represent the best‐fit lines from simple linear regression. The slope of the regression was statistically significant for both materials (*p*‐value < 0.0001). Sample sizes are *n* = 153 (ESCG) and *n* = 658 (HOPG). (c) Size‐dependent analysis of ESCG c‐axis electrical conductivity, with red circles representing data obtained from (a) at *h* =  908 nm (*n* = 70). The relationship was analyzed by simple linear regression, and the slope was statistically significant (*p*‐value = 0.003). Inset: Quantitative decomposition of area (yellow grid) and edge (purple solid line) contributions to total electrical conductivity, demonstrating the transition to area‐dominated transport (68%) above 2 µm. (d) Comprehensive comparison of our measured ESCG c‐axis resistivity with previously reported experimental values from NG and KG, with two theoretical estimated values denoted as dashed lines.

Based on extensive measurements, we report a conclusive value for the c‐axis resistivity of single‐crystal graphite: 5.7 ± 0.1 × 10^−5^ Ω · m, derived from hundreds of experimental data points on ESCG pillars, as shown in Figure 2a. This value was derived from four sets of ESCG pillars, fabricated with varying heights and multiple lateral dimensions to ensure a comprehensive dataset. The pillar height of each set was determined using white‐light interferometry (Figures S4 and S5 and Table S1). The data, summarized in Figure 2a, demonstrate compliance with Ohm's law:

(1) $$r_{t}=R\cdot S=\rho_{c}\cdot h+r_{\mathrm{int}}$$

where *r*_t_, *R*, ρ_c_ and *r*_int_ represents the specific resistance, measured resistance, c‐axis resistivity, and Pt/graphite interface resistance, respectively, *S* and *h* are the cross‐sectional area and height of the graphite layer. From these measurements, we determined the intrinsic c‐axis resistivity of ESCG, and the Pt/graphite interface resistance to be approximately 16.6 ± 0.8 Ω · µm^2^. Parallel electrical measurements for HOPG pillars (Figure 2b) revealed an average c‐axis resistivity of 1.91 ± 0.02 × 10^−3^ Ω · m, which is approximately two orders of magnitude higher than that of ESCG. Additionally, HOPG exhibited substantially greater data dispersion, reflecting its internally disordered structure. We also unified the axis scale of Figure 2a,b in Figure S14 for direct comparison.

To address concerns regarding edge effects in our microscale measurement system—a consideration relevant to frictional dissipation [43, 44, 45] and electrical transport [46, 47] we analyzed the size dependence of experimental data for the 900 nm height ESCG pillar set. The c‐axis electrical conductance of graphite can be decomposed into interior and edge contributions:

(2) $$\frac{h}{R_{c}}=\sigma_{\mathrm{in}}\;\cdot \left(S-P\cdot w\right)+\sigma_{\mathrm{edge}}\cdot P\cdot w$$

where $R_{c}=R-\frac{r_{\mathrm{int}}}{S}$ represents the c‐axis resistance of the graphite, *P* is the edge perimeter, *w* is the equivalent edge width, and σ_in_, σ_edge_ are the interior and edge conductivities, respectively. Further considering the square shape of graphite sample used, we introduce its side length *L* to obtain *P*  =  4*L* and *S*  = *L* ^2^ . For analytical simplicity, we linearized the equation:

(3) $$\frac{h}{R_{c}\cdot S}=\sigma_{\mathrm{in}}+\left(\sigma_{\mathrm{edge}}-\sigma_{\mathrm{in}}\right)\cdot w\cdot \frac{4}{L}$$

Linear fitting based on Equation 3 yielded values for σ_in_ =  16.51  ±  0.63  × 10^−3^ S · µm^−1^ and (σ_edge_ − σ_in_) · *w*  =  2.53 ± 0.82  × 10^−3^ S.

Substituting these parameters back into the original Equation 2 revealed the relative contributions of interior (yellow grid) and edge (purple solid line) components, as shown in the inset of Figure 2c. Due to the quadratic scaling of interior conductance with size compared to the linear scaling of edge conductance, interior transport becomes dominant (68%) at characteristic sizes exceeding 2 µm, as evidenced by the order‐of‐magnitude difference between the yellow dashed line and purple line in Figure 2c inset. However, the atoms on the edge indeed have much better conductivity. Taking the edge width 5 nm measured by Koren et al. [47], we could estimate the edge conductivity of Bernal stacking around 522.51 ± 164.63 S · µm^−1^, almost 1–2 orders of magnitude lager than the interior conductivity. (See Discussion S4 and Figure S6 for more details of the “electrical edge” of graphite micropillar.)

Comparing our results with previous experimental measurements and theoretical estimations of single‐crystal graphite c‐axis electrical resistivity (Figure 2d), we observe that earlier measurements on natural graphite and kish graphite show a noticeable variation in c‐axis resistivity. The data distribution suggests a correlation between increasing sample size and greater resistivity variation, potentially due to higher probabilities of defect occurrence. Furthermore, theoretical estimations from two different set of parameters yielded divergent values, contributing to uncertainty in fundamental understanding. Our comprehensive dataset of graphite c‐axis electrical resistivity at the micrometer scale, combined with Primak's results [19, 20] at the millimeter scale, presents a consistent result across more than three orders of magnitude in lateral scale.

The comparison between ESCG and HOPG resistance data (Figure 2a,b) revealed an intriguing phenomenon: HOPG exhibits increasing data dispersion with height, prompting a deeper investigation into the fundamental sources of graphite's c‐axis resistance based on our measured intrinsic resistivity values.

**FIGURE 3:**
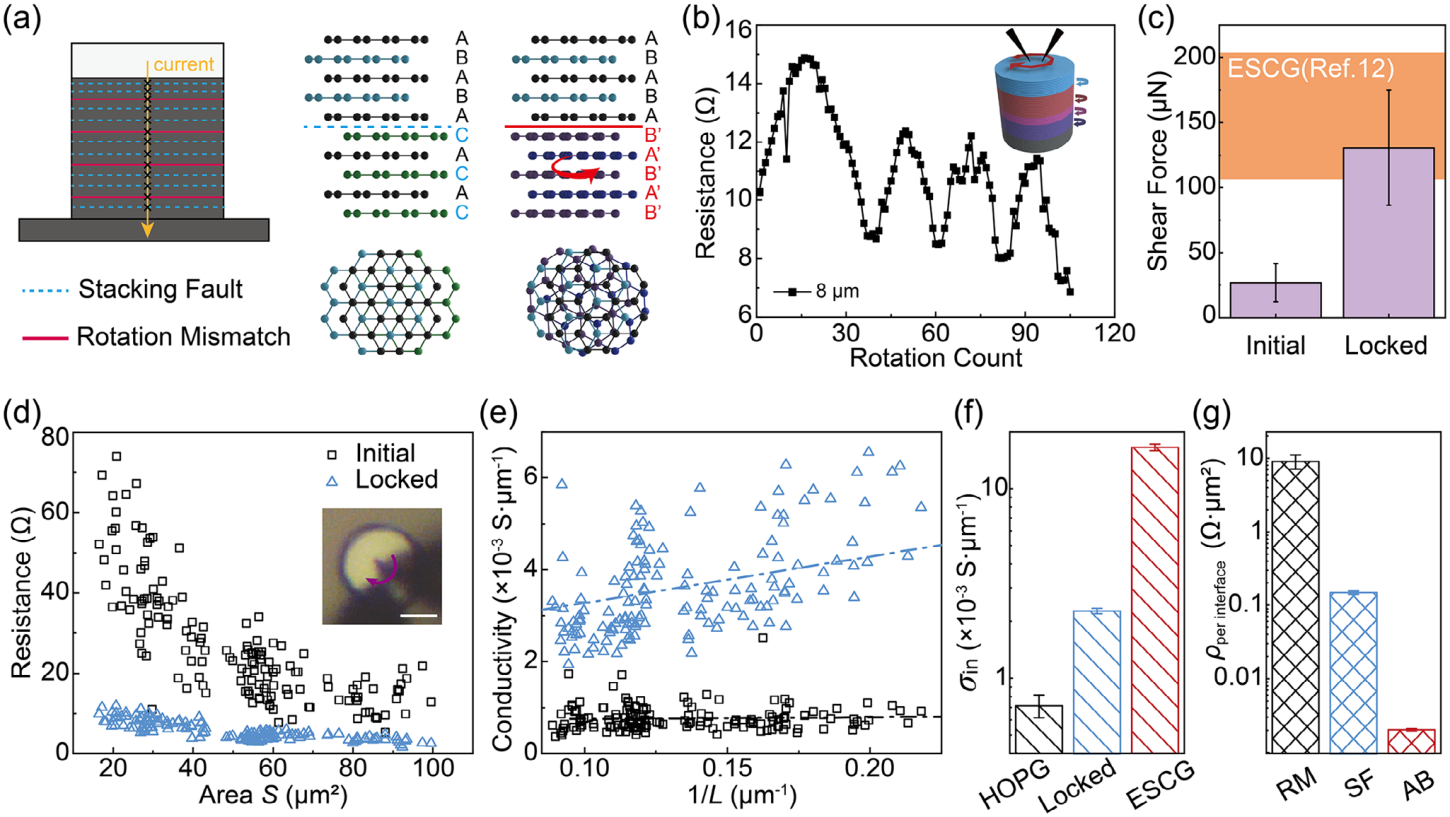
The origin of c‐axis electrical resistivity of graphite. (a) Structural representation of two primary c‐axis stacking configurations in graphite: SF (dashed blue line, middle panel) and RM (solid red line, right panel). (b) Continuous electrical resistance measurements during in situ unidirectional rotation, demonstrating the progressive transformation of incommensurate interfaces to commensurate locked states. Inset illustrates the sequential locking of internal incommensurate interfaces. (c) Mechanical verification of the completely locked state configuration (diameter, 8 µm). The shear force increased significantly in the locked state (130.8 ± 44.3 µN, *n* = 19) compared to the initial state (26.9 ± 16.8 µN, *n* = 10). (d) Comparative analysis of graphite resistance between the initial state (black squares) and final locked state (blue triangles) across various sample dimensions. Inset: Optical microscope snapshot during rotation, scale bar: 4* *µm. (e) Conductance per unit area (*h*/*R*) vs. 1/*L* for initial and locked states (*n* = 147). For the locked samples, the data were linear fitted with a fixed slope. For the initial samples, the relationship was analyzed by simple linear regression; the slope was not statistically significant (*p*‐value = 0.556), indicating the transport is area‐dominated. (f) Systematic comparison of c‐axis conductivity of HOPG, locked HOPG and ESCG. (g) Equivalent resistivity contribution from RM and SF, as well as the intrinsic AB stacked graphite resistivity.

From the perspective of c‐axis stacking, compared to the ideal AB stacking in ESCG, HOPG contains two additional primary defect types: SF which are commensurate interfaces, and RM which are incommensurate interfaces (Figure 3a). These defects differ significantly in their shear strength: RM interfaces exhibit structural superlubricity with a shear strength on the order of kPa [12, 43], markedly lower than that of SF and AB stacking on the order of 10 mPa [39]. This disparity enables selective mechanical manipulation of RM interfaces—via a rotational locking operation (see Figure S7), in which the RM interface is gradually rotated into a commensurate orientation. As an RM interface locks, newly exposed RMs hidden within the structure may emerge, and the process continues until all incommensurate interfaces are eliminated, as illustrated in the inset of Figure 3b. The operation terminates when no further rotation occurs, evidenced by the tungsten probe slipping on the Pt cap while the graphite flake remains fixed to the substrate, indicating complete locking. (The possible influence of in‐plane polycrystallinity is discussed in Discussion S5.)

We monitored the c‐axis resistance in situ during this rotational locking process. As shown in Figure 3b, the resistance exhibited oscillatory decreases, corresponding to the sequential locking of hidden RM interfaces. (See Figure S8 for more details.) The first RM interface initially at low twist angles (e.g., 15°) rotated through 30° before reaching a locked configuration at 60°, resulting in non‐monotonic resistance changes. Then the appearance of newly exposed RMs further contributed to these fluctuations, finally producing the characteristic oscillatory decrease observed in Figure 3b. Here, it should be noted that the rotation count refers to the operation count of rotation & measurement cycles, in which one electrical measurement is taken after each incremental rotation. To further confirm the removal of RM interfaces, we conducted mechanical shear tests on the fully locked HOPG pillars. As shown in Figure 3c, the measured shear strength matches that of ESCG pillars reported by Yang et al. [12]. —with similar lateral dimensions and identical testing conditions—validating the absence of incommensurate interfaces. (The cross‐sectional STEM image of a locked HOPG pillar, shown in Figure S9, further confirms the elimination of incommensurate interfaces in the locked sample.)

This rotational locking operation thus enables in situ comparison of HOPG micropillars before and after RM elimination. Figure 3d shows the c‐axis resistance of both initial and locked states across various lateral sizes. After locking, the resistance decreases by 1–2 orders of magnitude, and the data dispersion is significantly reduced, confirming that the internal RM interfaces—distributed with random misfit angles—are the primary source of both high resistance and variability in HOPG. (A detailed discussion of the influence of off‐axis alignment on the locked‐state resistance is provided in Discussion S6.) Using the same analytical method employed in Figure 2c, we plotted conductivity as a function of inverse pillar diameter (1/*L*) in Figure 3e. Still, for the HOPG sample, interior conductivity dominates, without obvious edge relationship, thus we obtained its c‐axis conductivity of 0.72 ± 0.10 × 10^−3^ S · µm^−1^; while for the locked HOPG sample, considering the same edge of Bernal stacking, we exploit the edge contribution from ESCG measurement to obtain the locked HOPG c‐axis interior conductivity of 2.26 ± 0.08 × 10^−3^ S · µm^−1^, as shown in Figure 3f.

We propose a simplified model where ESCG represents ideal graphite with perfect AB stacking, locked HOPG contains only SFs, and pristine HOPG includes both SFs and RMs, as discussed in detail in Discussion S7. Treating the system as a series connection of discrete interfaces, we estimated the relative resistivity of RM, SF, and AB stacking contribution to be 956: 381: 60 Ω · µm. Based on the stacking densities of RM (1/95 nm^−^^1^, see Figure S10) and SF (1/3.9 nm^−^^1^, estimated by Koren et al. [33].), we further deduced the effective c‐axis resistivity per interface type of RM:SF:AB = 4507:74:1 (Figure 3g). Notably, despite their relatively sparse occurrence, RM interfaces dominate the overall c‐axis resistance, with SFs contributing to a lesser extent.

**FIGURE 4:**
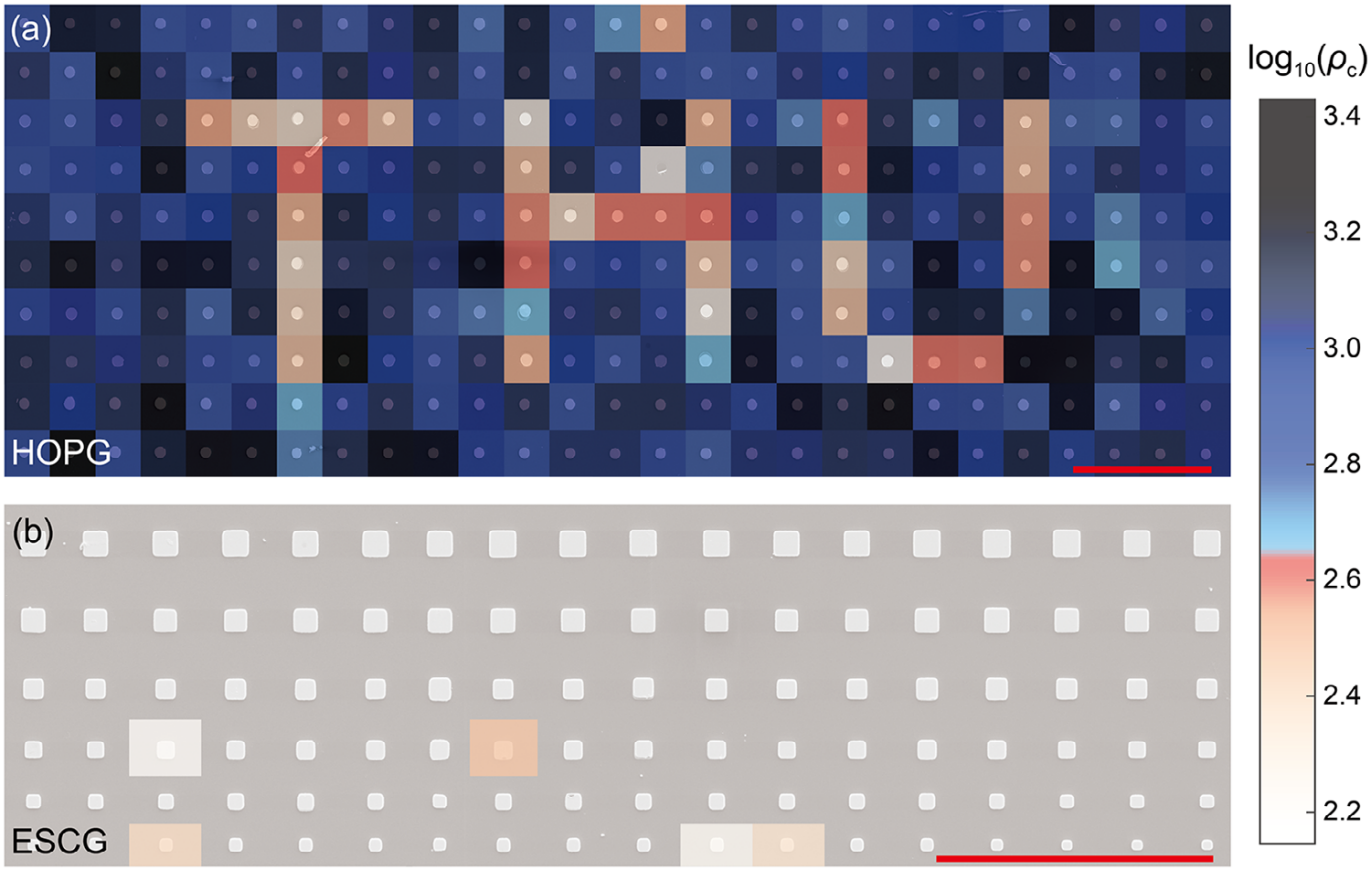
Pixelated visualization of electrical measurement distributions. (a) Spatial resistance mapping of HOPG showing values around 10^3^ Ω · µm. The red dashed region indicates the rotationally locked area, displaying an order of magnitude lower resistance and forming the “THU” pattern. Scale bar, 100* *µm. (b) Resistance mapping of ESCG demonstrating consistently low values around 10^2^ Ω · µm, indicating superior homogeneity across the measured area. The unit of resistivity *ρ*_*c*_ is Ω · µm. Scale bar, 100* *µm.

Furthermore, we developed a pixel‐array‐based visualization technique that offers a unique advantage in mapping the large‐scale lateral distribution of c‐axis conductivity in selected materials. This approach enables the detection of internal interfacial defects and facilitates mechanically induced tuning of electrical transport properties, as shown in Figure 4. (1) The ESCG sample exhibits a uniformly white across the mapped region, indicative of low and homogeneous resistance, consistent with its ideal AB stacking. A few regions exhibiting slightly elevated resistivity are likely due to microfabrication artifacts, such as anomalous interfacial resistance between the Pt cap and graphite. (2) In contrast, the HOPG sample shows markedly higher resistance with larger spatial fluctuations, reflecting the presence of internal SF and RM. (3) Leveraging the mechanical tunability of incommensurate interfaces, we further achieved targeted structural manipulation through rotational locking. This is vividly demonstrated by the emergence of a distinct “THU” pattern in the center of the HOPG array, where selectively locked regions exhibit a clear reduction in resistance.

The pixel‐array‐based visualization technique developed in this work has strong potential to evolve into a powerful tool for internal structural characterization of micro‐ and nanoscale layered materials. Traditional methods, reliant on light or electron signals, are limited by shallow penetration depths (restricting structural insights to near‐surface layers and necessitating complex sample preparation), while also struggling to balance range and precision. In contrast, our approach is well‐suited for detecting interfacial structures buried within thick layered systems. It combines electrical transport analysis to infer internal structural features, rotational manipulation for deliberate structural control, and systematic micropillar fabrication integrated with a fully automated measurement system [48] to achieve both high precision and large‐scale lateral mapping. Additionally, future enhancements—such as the integration of alternating current and direct current (AC/DC) transport measurements—could extend the method's applicability to a broader range of thick‐layered materials and enrich the type of detectable information.

## 3 Discussions

While the ESCG we utilized is currently the highest‐quality graphite available, it susceptible to defects and imperfections, which contribute to some dispersion in our measurement results. Nonetheless, we believe that the findings presented herein are sufficiently robust for most practical applications, such as calibrating electron transport models of vdW layered materials.

Moreover, by employing a novel rotational locking method, we have for the first time quantitatively determined the effective c‐axis resistivity per interface type of RM:SF:AB = 4507:74:1. This breakthrough provides deeper insights into the origins of c‐axis resistivity in graphite. However, the spatially varying distributions of SF and RM across different materials present ongoing challenges for rapidly and accurately determining bulk resistivity values. Looking forward, our proposed arrayed‐pixel controllable resistor concept may potentially evolve into a novel class of mechanically controlled electronic devices in future applications.

Notably, phonon contributions play a key role in determining the c‐axis transport in graphite under the room‐temperature conditions of our study. Future low‐temperature experiments on this system may reveal unique quantum phenomena that emerge when phonons are frozen out, such as coherent transport occurring only at specific commensurate twist angles [34]. A preliminary discussion on this point is provided in Discussion S8.

## 4 Methods

### 4.1 Preparation of Graphite Micropillar

Graphite micropillar arrays with a Pt cap were fabricated on HOPG and ESCG, following the process detailed in Figure S1. The fabrication procedure involved the following steps: (1) Mechanical exfoliation was performed on HOPG or ESCG to expose a freshly cleaved, smooth surface for subsequent processing; (2) A layer of photoresist was spin‐coated onto the graphite surface; (3) Electron beam lithography was used to selectively remove photoresist in predefined regions, defining the array pattern for the graphite micropillars; (4) A Pt film was deposited via electron beam evaporation, with a titanium layer as an adhesion layer to promote bonding between the graphite and Pt; (5) A lift‐off process was carried out to define the Pt pattern array; (6) Graphite micropillar arrays were formed by reactive ion etching (RIE), using the Pt cap as an etching mask.

### 4.2 Four‐Wire Resistance Measurement

All electrical measurements were conducted using tungsten probes controlled by a micromanipulator (MM3A, Kleindiek). A four‐wire measurement configuration was employed, utilizing a source meter (Keithley 2450). Two terminals were connected to the HOPG substrate, while the remaining two were connected to tungsten probes for current injection and voltage sensing, as shown in Figure 1d. The corresponding equivalent circuit is illustrated in Figure S2. First, both probes were placed on the Pt cap to measure the voltage *V*_1_. Then, the voltage‐sensing probe was moved to the graphite substrate to measure *V*_2_ (as indicated by the white dashed box in Figure 1d). The c‐axis resistance of the graphite micropillar (*R*_cap_+*R*_pillar_) was obtained by calculating (*V*_1_ – *V*_2_)/*I*.

### 4.3 Statistical Analysis

The original resistance data were directly obtained from graphite pillar arrays using a Keithley 2450. Each data point in the scatter plot represents a single measurement on an independent pillar. All data points were retained for analysis, as they fell within the expected physical range and exhibited good reproducibility. For measurements of the locked‐state resistance in Figure 3, data from pillars that failed to reach the locked state were excluded from statistical analysis. Values in the text are reported as mean ± SD unless reported otherwise. The relationships between resistance and sample height, as well as between edge and interior decoupling, were modeled using linear regression according to Equation 1 and 3, implemented via the least‐squares method. The statistical significance of the regression was assessed by testing the null hypothesis that the slope of the regression line is equal to zero. The two‐tailed *p*‐value associated with the slope's t‐statistic is reported in the figure legends. A *p*‐value of less than 0.05 (p < 0.05) was considered statistically significant. All data processing, regression analysis was performed using Origin and MATLAB.

## Conflicts of Interest

The authors declare no conflict of interest.

## Supporting information




**Supporting File**: advs73713‐sup‐0001‐SuppMat.docx.

## Data Availability

The data that support the findings of this study are openly available in Data for Origins of Graphite Resistivity: Decoupling Stacking Fault and Rotational Misorientation at https://doi.org/10.5281/zenodo.17461005, reference number 49.
