# Supplementary material for: Origins of Graphite Resistivity: Decoupling Stacking Fault and Rotational Misorientation
Source: Adv Sci (Weinh). 2026 Jan 7;13(16):e18254. doi: 10.1002/advs.202518254 (PMC13042675; doi:10.1002/advs.202518254)
Supplement: Supplementary file 1 — Supporting File: advs73713‐sup‐0001‐SuppMat.docx. [file ADVS-13-e18254-s001.docx]

Supporting Information

Origins of Graphite Resistivity: Decoupling Stacking Fault and Rotational Misorientation

*Weipeng Chen, Fuwei Yang, Tielin Wu, Yelingyi Wang, Quanshui Zheng, Deli Peng*, and Zhanghui Wu**

**Table of Contents**

| **Supplementary Figure S1-S12**   1. Fabrication process of graphite micropillar array 2. Equivalent electrical circuit diagram 3. Robust electrical resistance measurement: no observable pressure dependence 4. Height measurement using white-light interferometry 5. Supplementary information of the pillar height in Figure 2a&2b 6. Comparison of electrical resistance before and after etching the physical edge of amorphous carbon 7. Rotational “lock” operation for eliminating incommensurate interfaces based on a dual-probe method 8. The resistance evolution during continuous rotational locking process 9. Cross-sectional STEM characterization of the locked HOPG pillar. 10. The stacking density of RM in HOPG 11. Influence of the size and position of contact pad 12. High-temperature experiment on micro-scale graphite pillars 13. Low-temperature measurements on bulk graphite samples 14. Finite element simulation of electrical transport in bulk samples 15. SEM image of the locked HOPG pillar 16. Combination of Fig. 2a&2b for direct comparison 17. Laue diffraction pattern | Pgs.3-10 |
| --- | --- |
| **Table S1 \|** Statistic of the pillar height | Pg.11 |
| **Supplementary Discussion 1: Influence of contact pad size and position** | Pg.12 |
| **Supplementary Discussion 2: Investigation of phonon contributions via high-temperature substitution experiments** | Pg.12 |
| **Supplementary Discussion 3: Low-temperature measurements on bulk samples and consistency with historical studies** | Pg.13 |
| **Supplementary Discussion 4: The “electrical edge” of graphite micropillar** | Pg.14 |
| **Supplementary Discussion 5: Assessing the possible influence of in-plane polycrystallinity** | Pg.14 |
| **Supplementary Discussion 6: The influence of off-axis alignment on the locked-state resistance** | Pg.15 |
| **Supplementary Discussion 7: The stacking structures of ESCG, HOPG, and locked HOPG** | Pg.15 |
| **Supplementary Discussion 8: Phonon contributions and their implications for low-temperature transport** | Pg.16 |
| **References** | Pgs.18 |


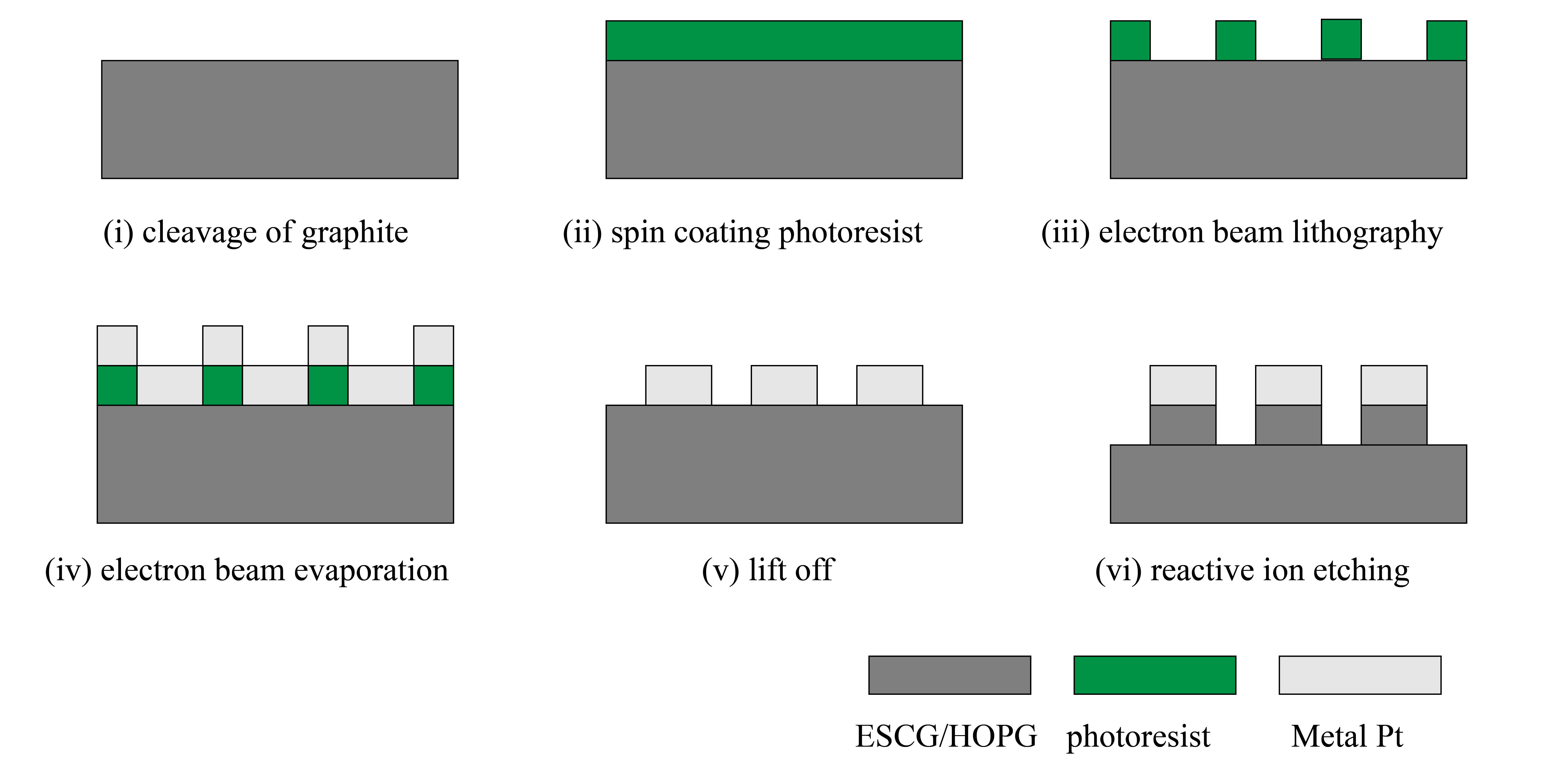


**Figure S1. Fabrication process of graphite micropillar array.** (i) A pristine, atomically flat graphite (ESCG/HOPG) surface was prepared via mechanical exfoliation. (ii) A photoresist layer was spin-coated uniformly onto the surface. (iii) Electron beam lithography was employed to define the flake array pattern by selectively removing the photoresist. (iv) A Pt film was deposited by sputtering, with a titanium adhesion layer to promote bonding between the Pt and graphite. (v) The Pt pattern array was formed via a lift-off process. (vi) Graphite flake arrays were then obtained by reactive ion etching, using the patterned Pt caps as hard masks.


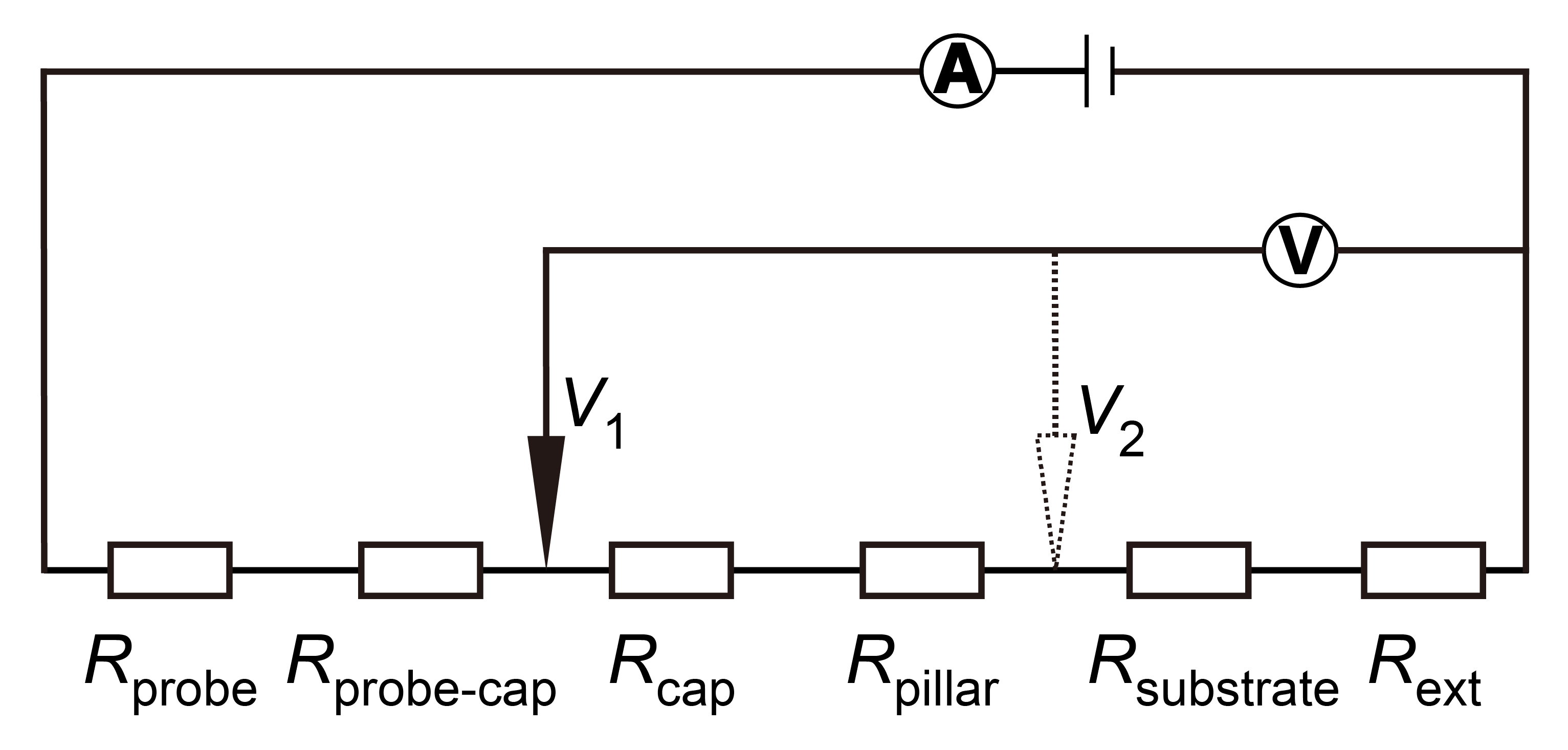


**Figure S2.** **Equivalent electrical circuit diagram.** *R*_probe_, *R*_probe-cap_, *R*_cap_, *R*_pillar_, *R*_substrate_, and *R*_ext_ represent the resistance of the current-source probe (probe 1), interface between probe 1 and cap, Pt cap, graphite pillar, graphite substrate and external circuit, respectively. The voltmeter probe (probe 2) was pressed first on the Pt cap (black solid) and then on the graphite substrate (white dashed line), consistent with Figure 1d, to get corresponding voltages *V*_1_ and *V*_2_.


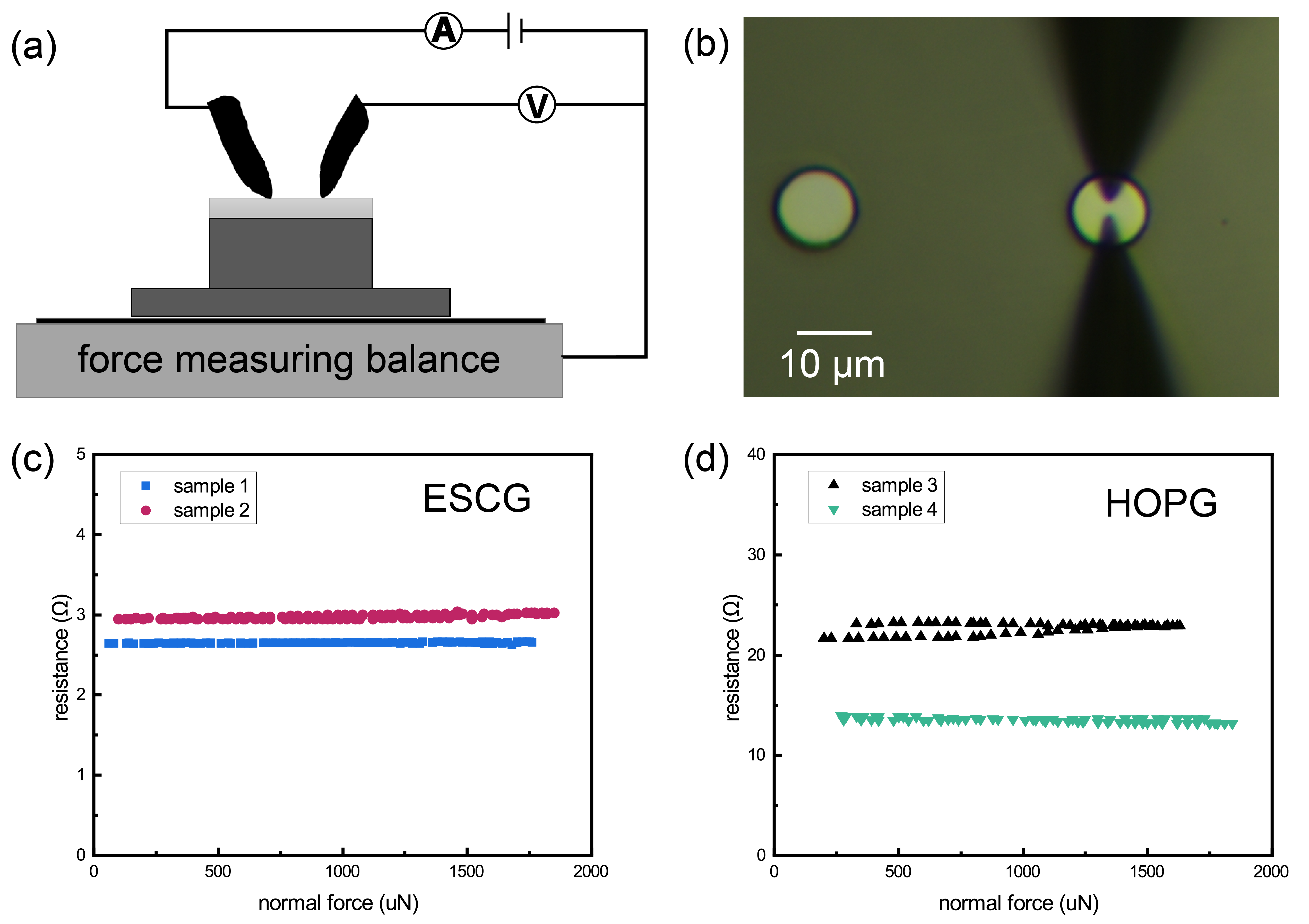


**Figure S3. Robust electrical resistance measurement: no observable pressure dependence.** (a) Schematic diagram, with the graphite sample measured on force measuring balance. (b) Optical snapshots for the resistance 𝑅_1_ and 𝑅_2_ measured with the voltmeter probe on the flake cap and graphite substrate. (c)&(d) Measured resistance versus normal force applied on the graphite flake, with no observable pressure dependence in either ESCG or HOPG. I = 5 mA.


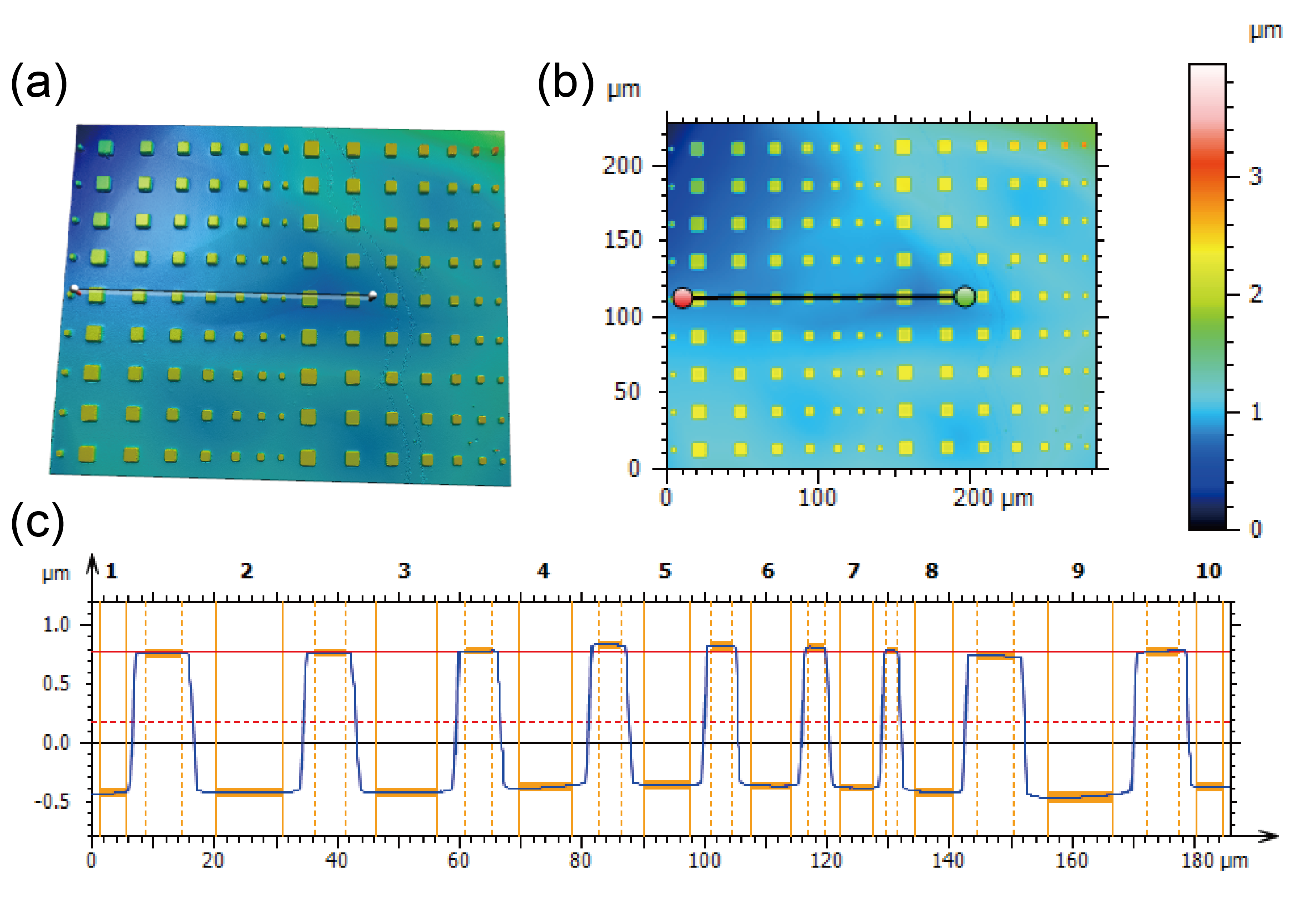


**Figure S4. Height measurement using white-light interferometry.** (a) Optical image of the selected region. (b) Corresponding height map obtained via white-light interferometry. (c) Height profile along the selected line in (b), revealing consistent pillar height across the measured area.

**
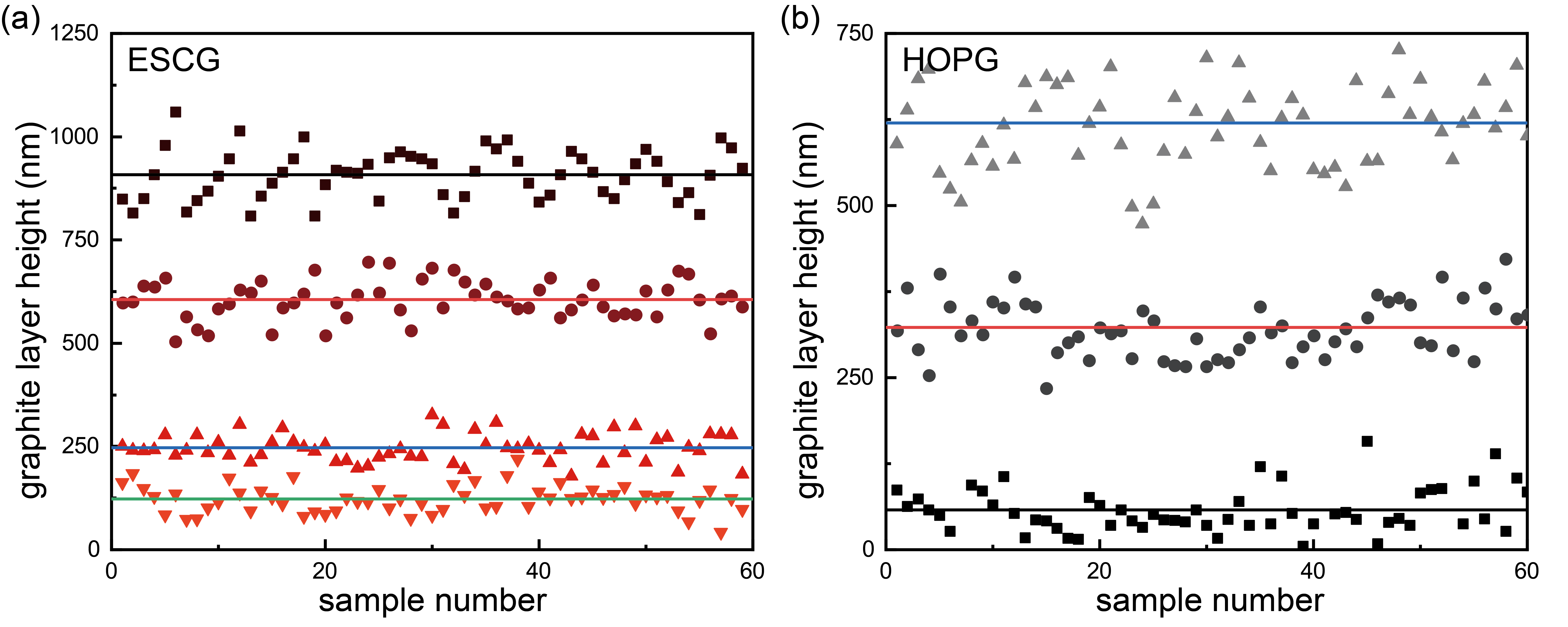
**

**Figure S5. Supplementary information of the pillar height in Figure 2a&b.** (a) ESCG. (b) HOPG. Statistical results are as detailed in Supplementary Table S1.


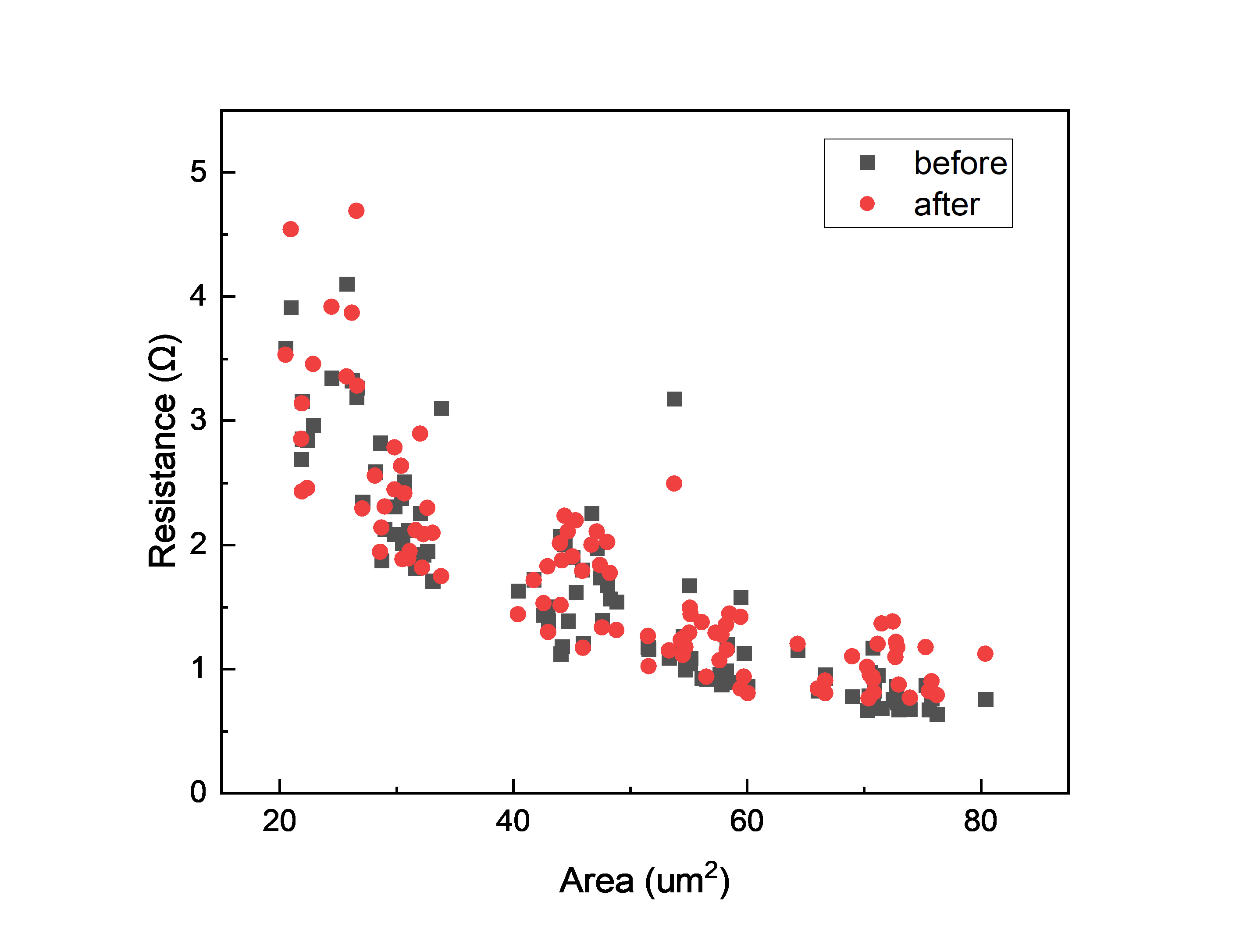


**Figure S6. Comparison of electrical resistance before and after etching the physical edge of amorphous carbon.** The results show negligible change in resistance following the removal of amorphous carbon at the pillar edge, indicating that the physical edge does not significantly contribute to c-axis electrical transport.


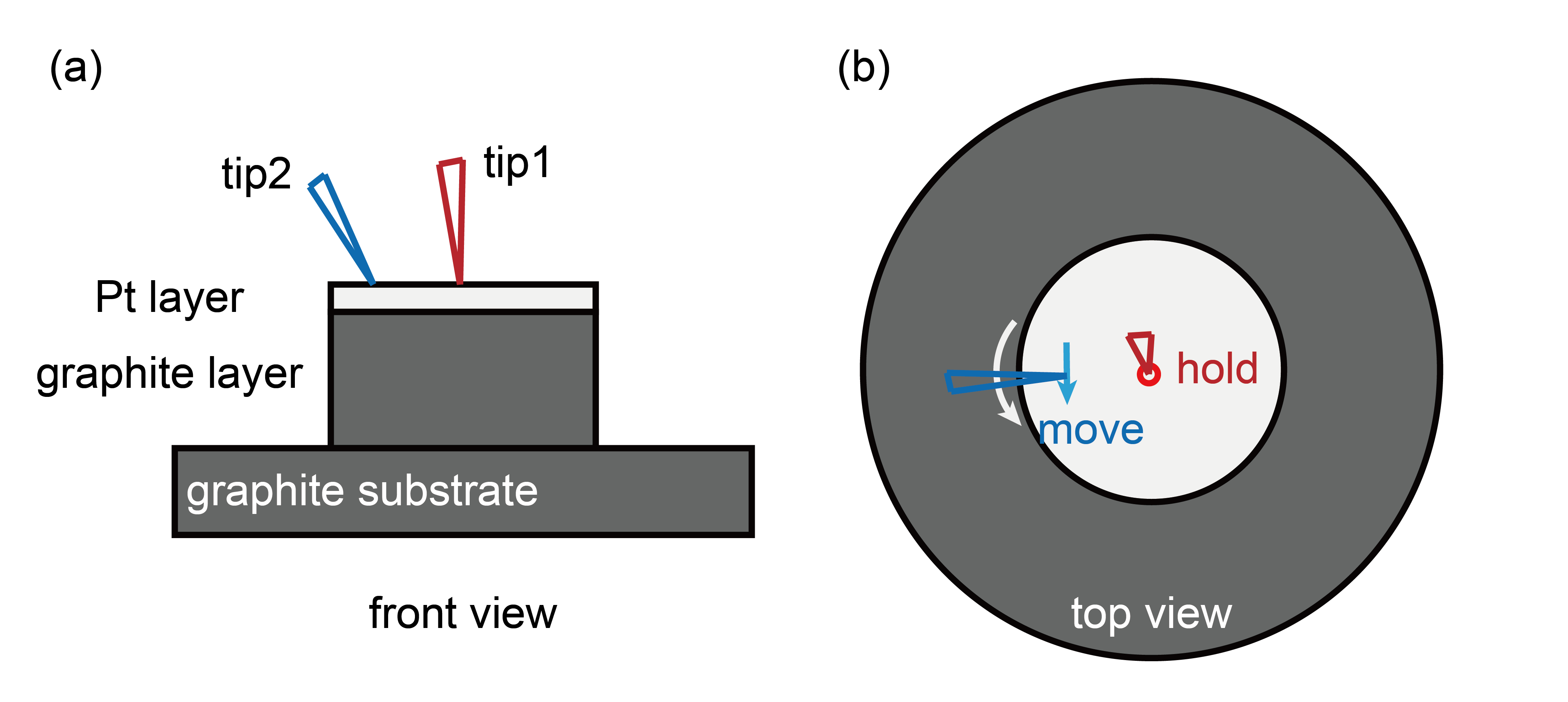


**Figure S7. Rotational “lock” operation for eliminating incommensurate interfaces based on a dual-probe method.** (a) Front view of the dual-probe setup, where two tungsten micro-tips are positioned on the Pt cap of a graphite micropillar. (b) Top view illustrating the rotational locking procedure: Tip 1 is placed at the center of the pillar to anchor it, while Tip 2 is positioned near the edge and moved laterally by ~1 μm to induce rotation. After each displacement, Tip 2 is lifted and returned to its original position before repeating the process. Due to the 60° periodicity of incommensurate graphite interfaces, this method incrementally eliminates rotational mismatches until a commensurate (locked) state is achieved.

**
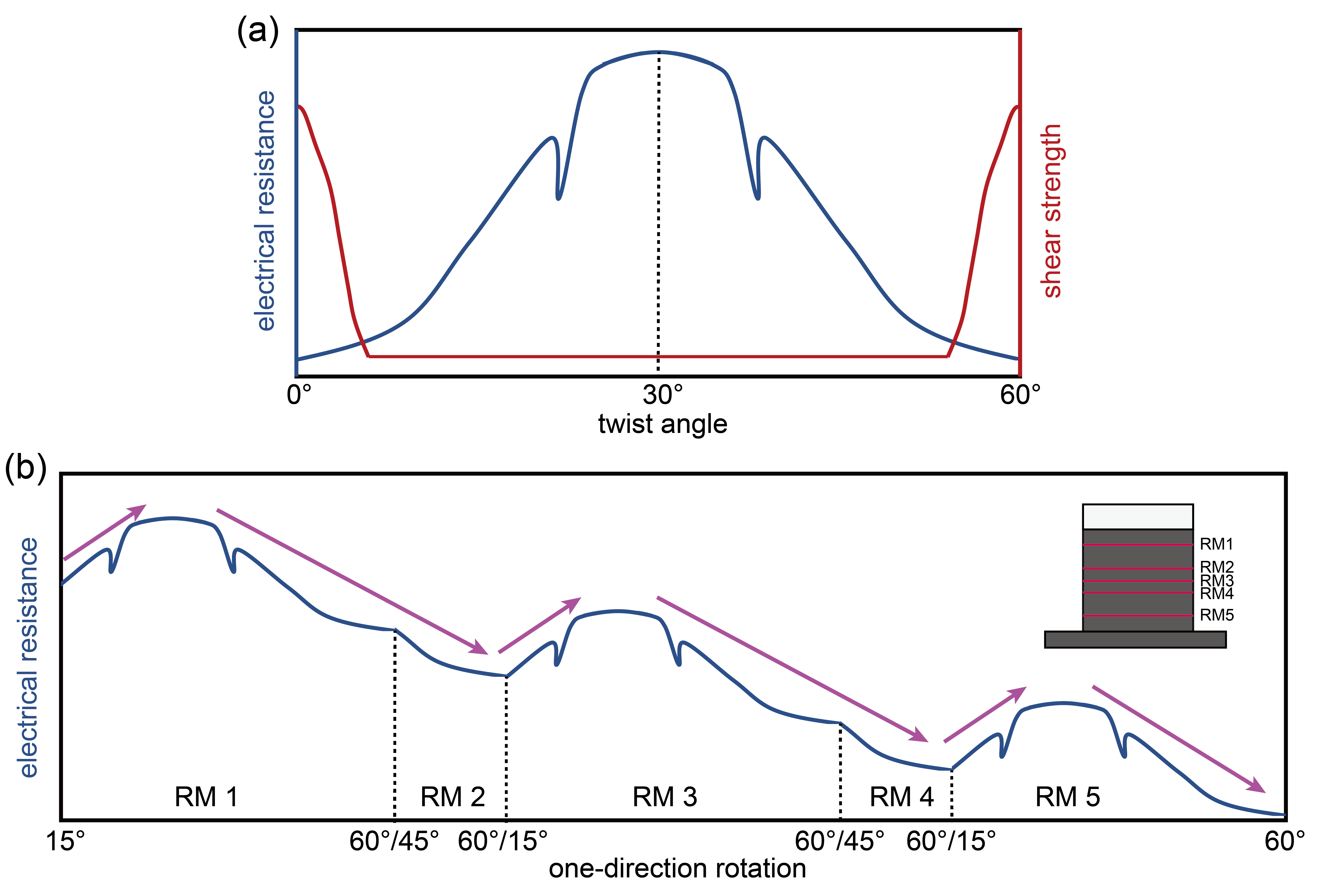
**

**Figure S8. The resistance evolution during continuous rotational locking process.** (a) Schematic illustration showing the dependence of electrical resistance (blue curve) and shear strength (red curve) on the twist angle, adapted from Koren et al.^1^ and Dienwiebel et al.^2^ (b) Simulated resistance evolution and interfacial transitions during the continuous rotational locking process. Two representative initial RM twist angles, 15° and 45°, are considered, finally reproducing the experimentally observed oscillatory decreases shown in Figure 3b.


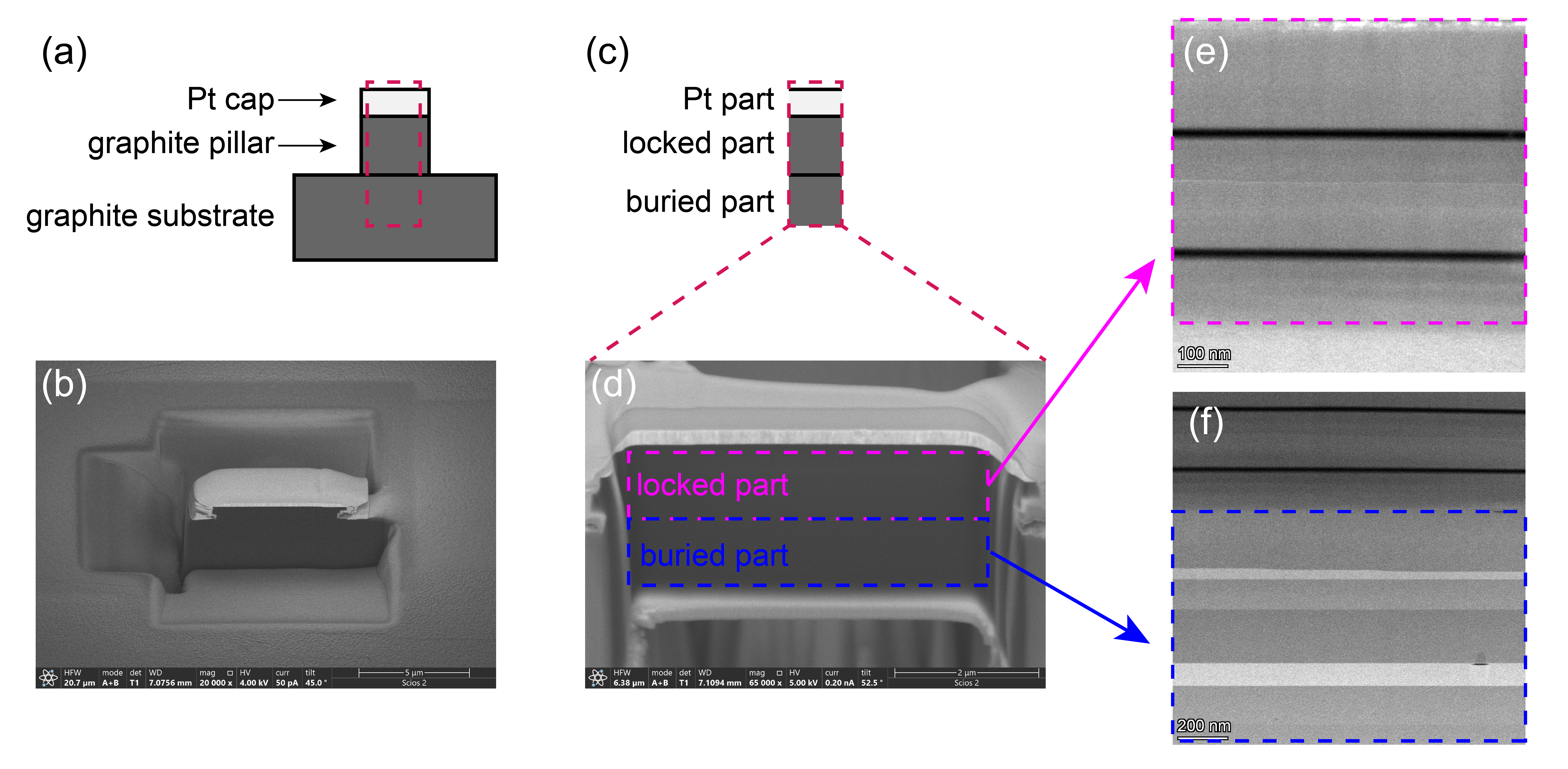


**Figure S9. Cross-sectional STEM characterization of the locked HOPG pillar.** (a) Schematic illustration of the graphite pillar on the graphite substrate, with the red dashed box indicating the region extracted by FIB. (b) Cross-sectional view of the corresponding graphite pillar. (c) Structural schematic of the FIB-prepared lamella, including the Pt cap, locked graphite layers, and the buried pristine graphite substrate. (d) SEM image of the FIB-cut lamella corresponding to (c). (e) Cross-sectional STEM image of the locked graphite region, exhibiting uniform contrast that indicates coherent structural alignment. (f) STEM image of the buried graphite region, showing contrast variations characteristic of HOPG domains separated by twist-angle grain boundaries. The two dark bands observed in panel (e) are attributed to minor mechanical cracks formed during FIB preparation due to thermal expansion mismatch between Pt and graphite; these do not affect the interpretation of the locked structure.


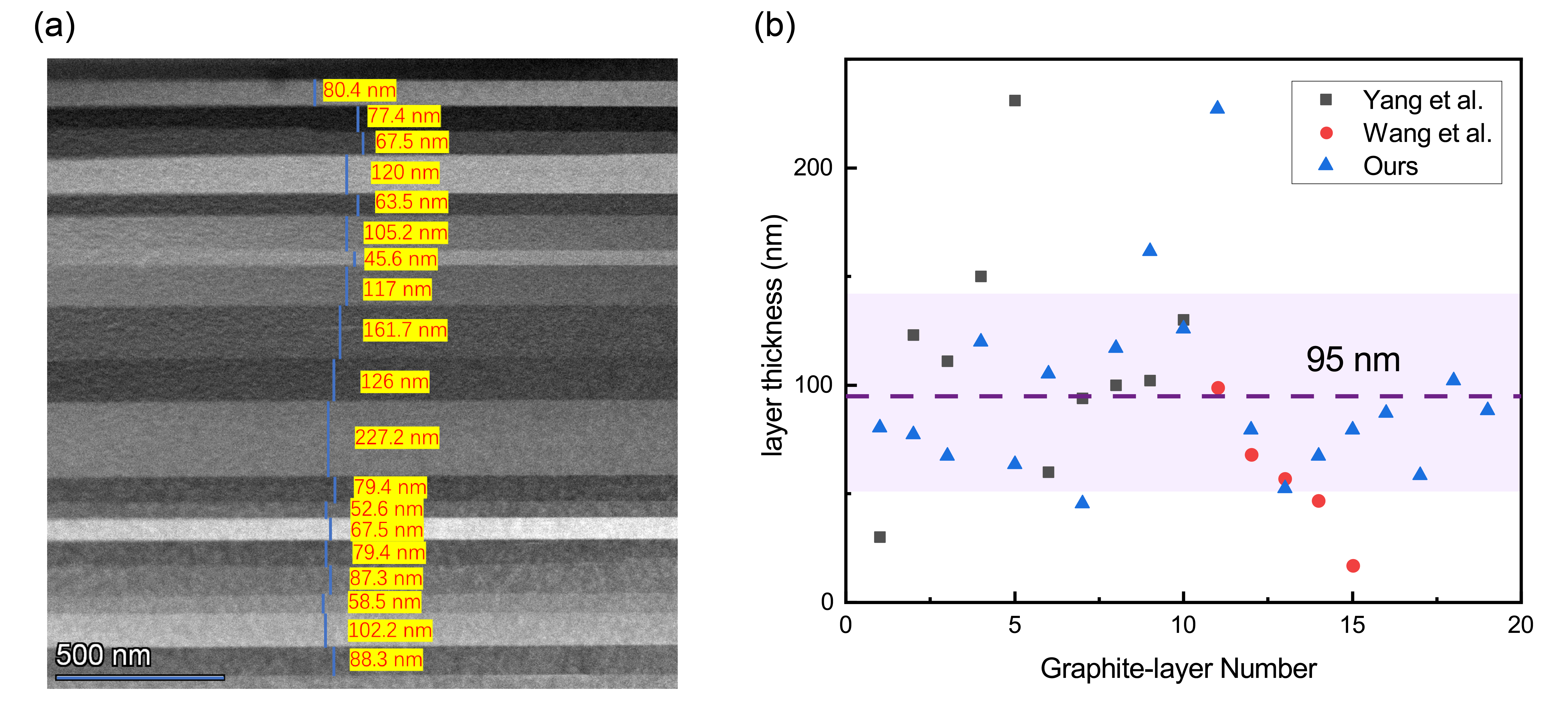


**Figure S10. The stacking density of RM in HOPG.** (a) Cross-sectional STEM image of HOPG, revealing randomly spaced RM interfaces that disrupt the ideal stacking sequence at intervals of several tens of nanometers. (b) Combined with previous characterization results reported by Wang et al.^3^ and Yang et al.^4^, a statistical distribution of RM interlayer spacings is obtained, yielding an average spacing of approximately 95 nm.


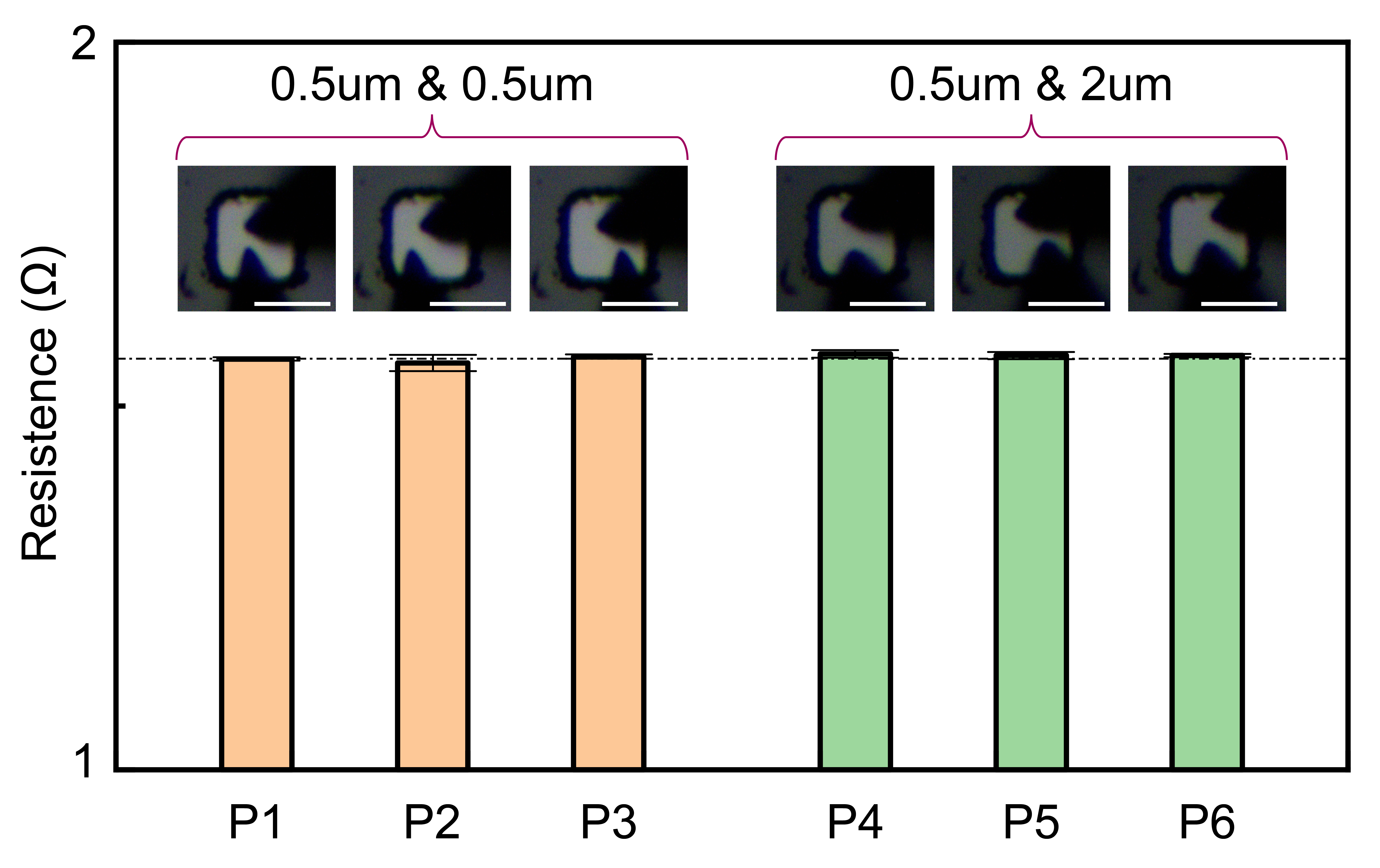


**Figure S11. Influence of the size and position of contact pad.** Two sets of probes with different diameters were used and their positions were varied across three measurement points; the measured resistances exhibited good consistency (1.5651 ± 0.0189 Ω). Scalebars, 9 μm.


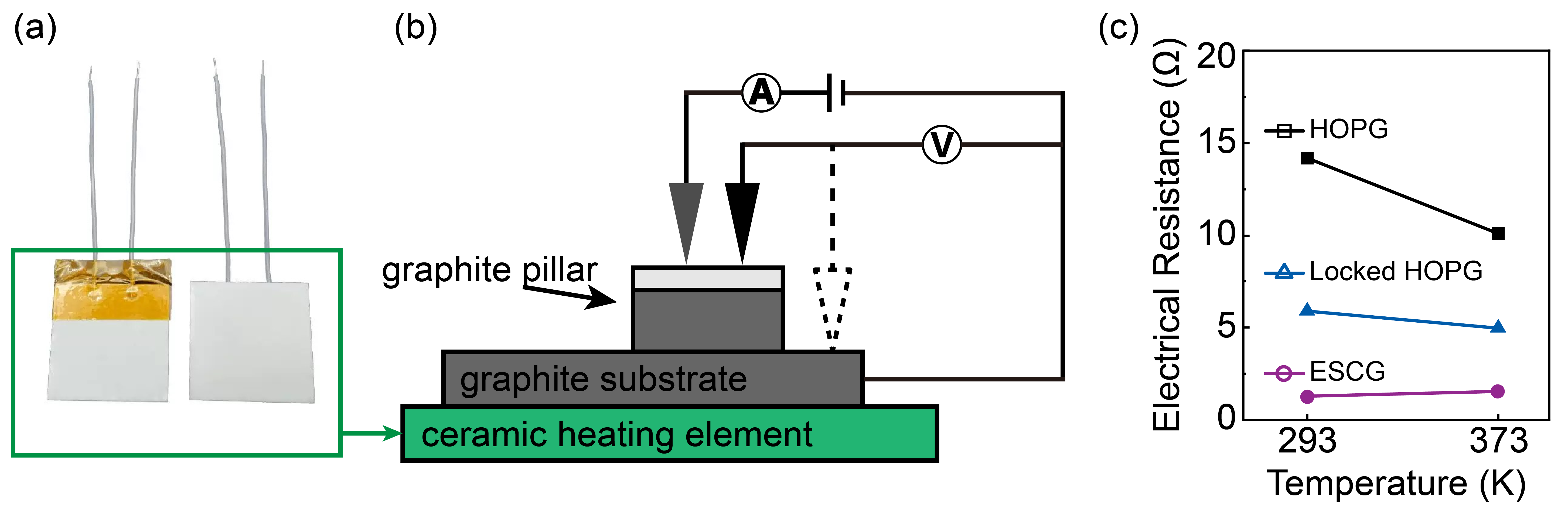


**Figure S12. High-temperature substitution experiment on micro-scale graphite pillars.** (a) Actual picture of MCH ceramic heater. (b) Schematic of the experimental setup. The graphite sample is placed on a ceramic heater, enabling resistance measurements at different temperatures (ON/OFF states). (c) Comparison of electrical resistance for three graphite samples (HOPG, locked HOPG and ESCG) at room temperature (293 K) and high temperature (373 K). The pillars of HOPG and locked HOPG have a diameter of 8 μm and thickness of 900 nm; the ESCG sample has a side length of 6.5 μm and height of 620 nm. Measurement current at 5 mA.


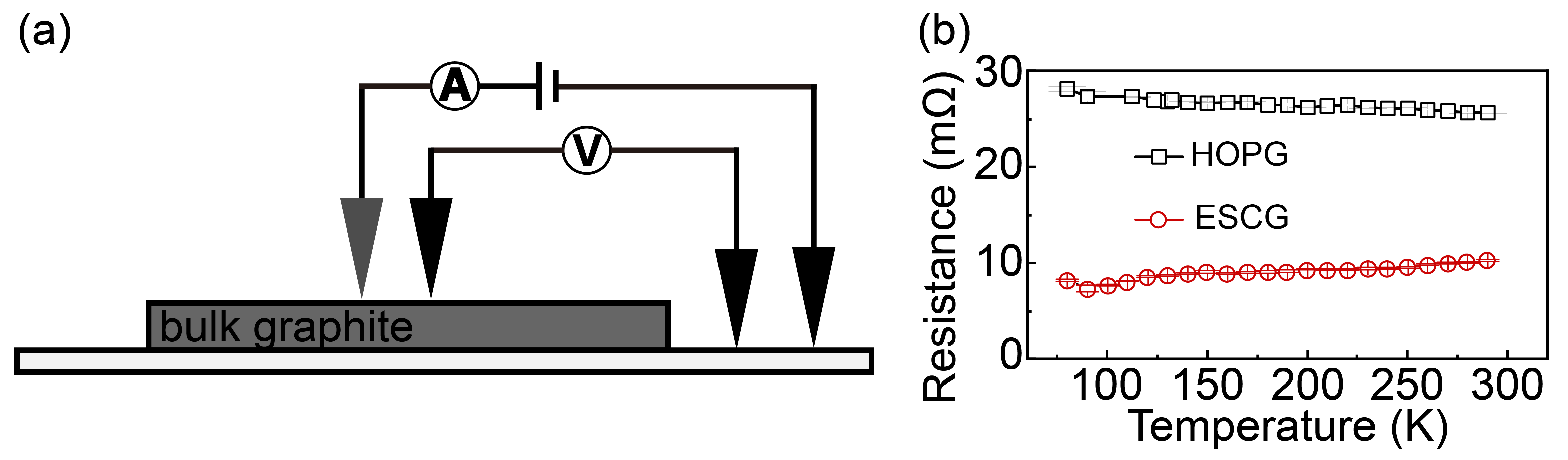


**Figure S13.** **Low-temperature measurements on bulk graphite samples.** (a) Schematic of the experimental setup. (b) Temperature-dependent resistance curves for bulk HOPG and bulk ESCG, showing opposite trends


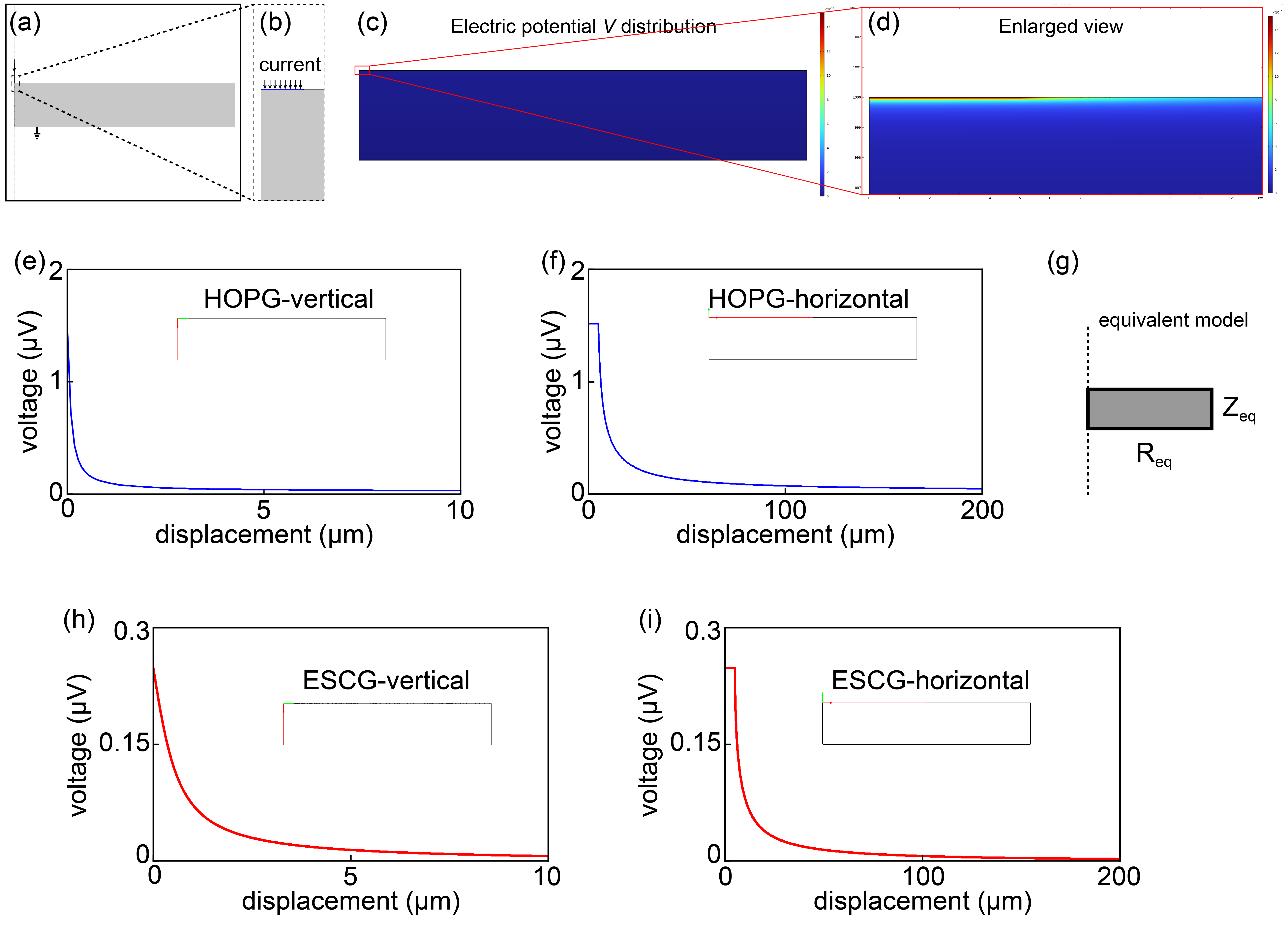


**Figure S14. COMSOL finite element simulation of electrical transport in bulk samples.** (a) 2D axisymmetric model setup. (b) Current injection simulation condition (*I* = 1 μA, radius 5 μm). (c, d) Potential distribution showing localization near the probe. (e, f) Potential drops along the axis of vertical symmetry (e) and horizontal direction of the surface (f), for bulk HOPG. (g) Schematic of effective measurement geometry. (h, i) Potential drops along the axis of vertical symmetry (e) and horizontal direction of the surface (f), for bulk ESCG.


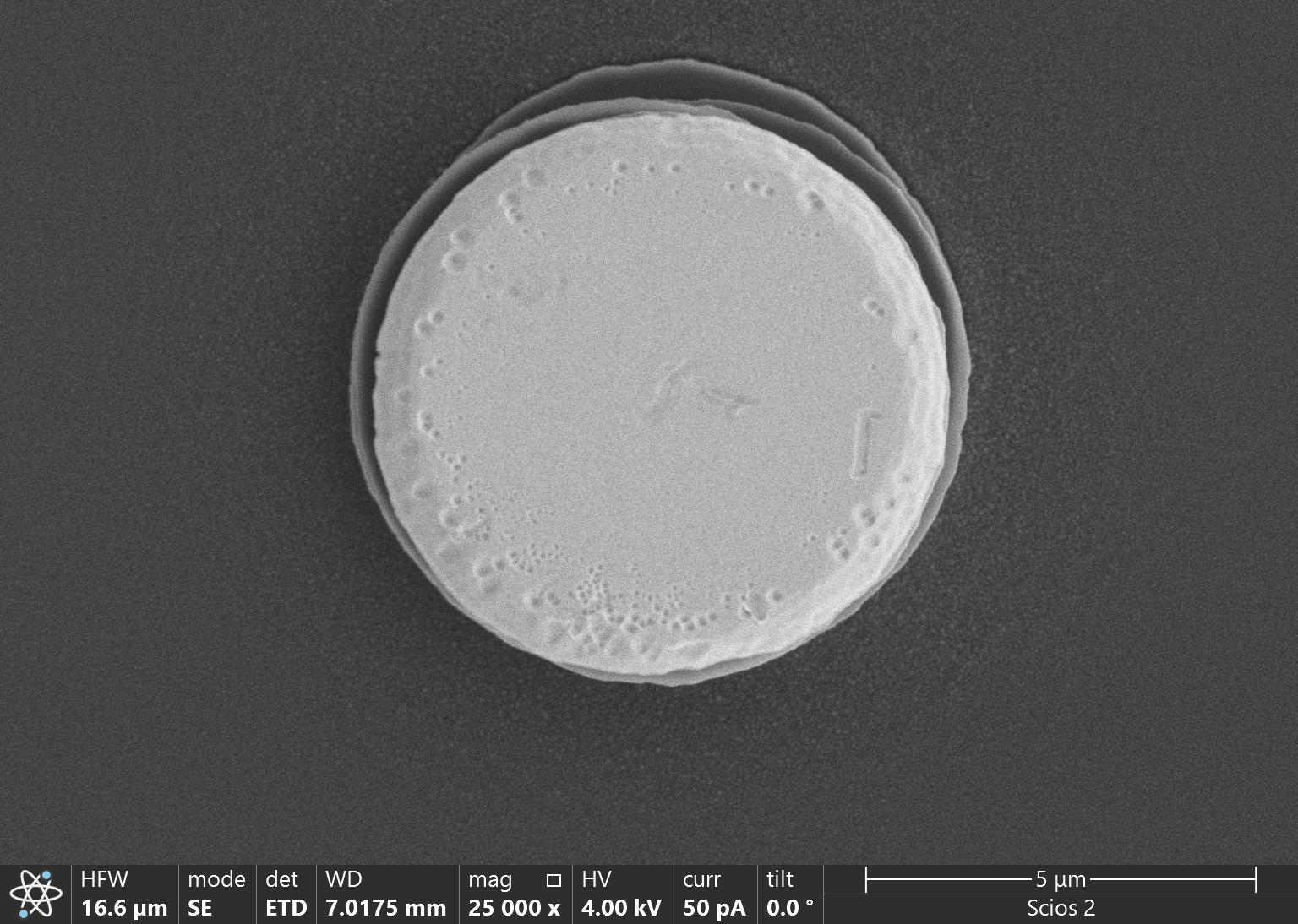


**Figure S15. SEM image of the locked HOPG pillar.**


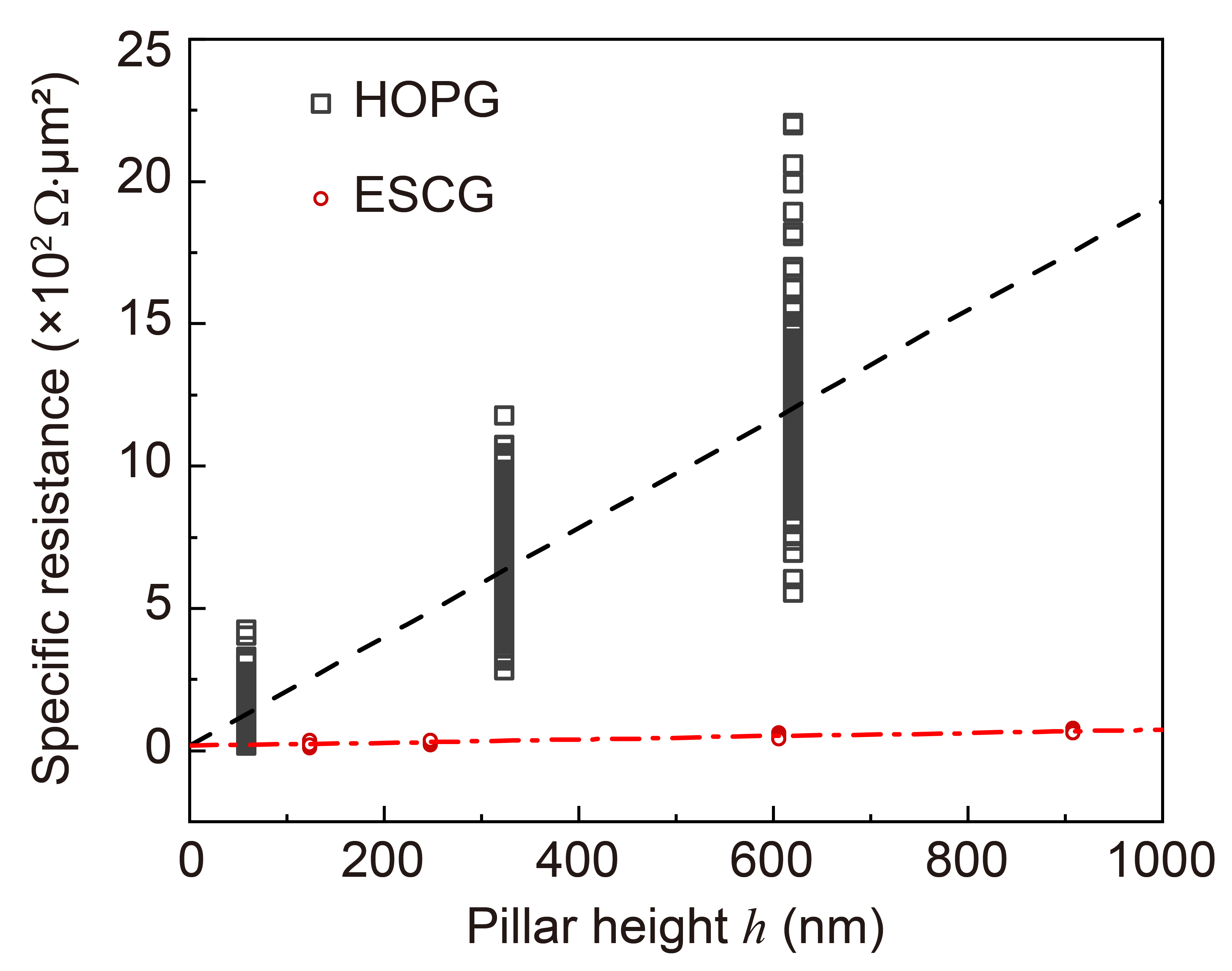


**Figure S16 | Combination of Figures 2a&2b for direct comparison.**


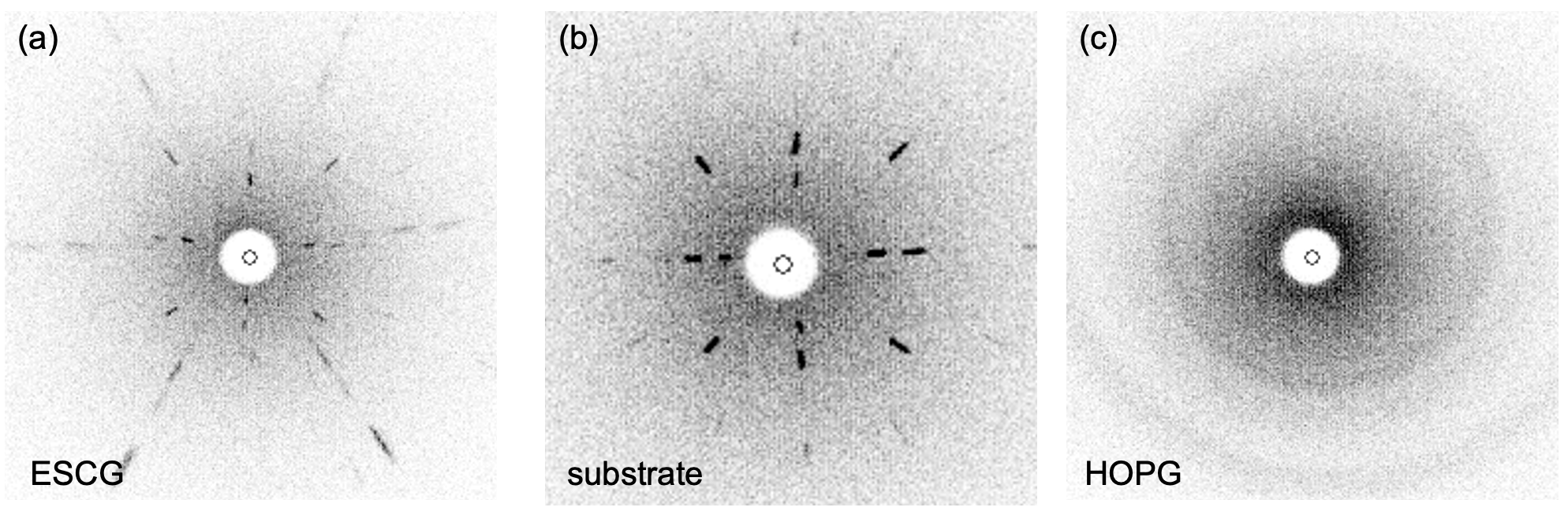


**Figure S17 | Laue patterns of the ESCG (a), Si substrate (b) and HOPG (c).** The diameter of the aperture is 1 mm.

**Table S1 | Statistic of the pillar height (not including Pt height).**

| Graphite Set | Mean (nm) | Standard Deviation (nm) |
| --- | --- | --- |
| ESCG | 907.75 | 58.87 |
|  | 605.49 | 46.9 |
|  | 246.8 | 33.82 |
|  | 122.24 | 32.83 |
| HOPG | 57.8 | 32.0 |
|  | 323.3 | 40.0 |
|  | 620.1 | 58.9 |

## Supplementary Discussion 1: Influence of Contact Pad Size and Position

Accurately evaluating the intrinsic c-axis resistivity at the microscale requires minimizing the effect of contact resistance, which becomes increasingly significant as the measurement area decreases. In our experiments, a Pt capping layer was deposited on top of each graphite pillar to serve as an equipotential surface, thereby reducing the sensitivity of the measurement to the contact state between the tungsten probes and the graphite. Two main types of contact resistance are considered:

1. Contact resistance between the tungsten probes and the Pt cap (contact pad): To minimize this contribution, we employed a two-probe configuration based on a four-wire measurement scheme, where one probe supplies current and the other measures the potential of the equipotential Pt cap. Benefiting from the high conductivity of Pt, this method ensures robust and reproducible measurements that are essentially independent of the probe size or contact position. As shown in Figure S11, two sets of probes with different diameters were used and their positions were varied across three measurement points; the measured resistances exhibited good consistency (1.5651 ± 0.0189 Ω). Set 1 used two tungsten probes of ST-20-0.5 (radius of 0.5 μm, GGB); and set 2 used one tungsten probe of ST-20-0.5 (radius 0.5 μm of GGB) and one tungsten probe of ST-20-2 (radius of 2 μm, GGB).
2. Contact resistance between the Pt cap and the graphite pillar: This contribution was quantitatively evaluated through height-dependent measurements, as shown in **Figure 2**a. Because all Pt caps were fabricated using the same deposition process, the intercept of the fitted linear relation provides a reliable estimation of the Pt–graphite contact resistance, yielding a value of (16.6 ± 0.8 Ω·μm²).

## Supplementary Discussion 2: Investigation of phonon contributions via high-temperature substitution experiments

Our study focuses on micro-scale graphite pillars (lateral dimensions <10 μm). Reliable four-probe electrical transport measurements on this scale require high-precision micro-manipulators (e.g., the Kleindiek system used in our main text) and a high-resolution optical system (×50 magnification) for assisted positioning. Standard commercial low-temperature vacuum probe stations are typically constrained by chamber size, offering only manual manipulators and low-resolution optical system (e.g., ×5 magnification). This makes contacting micro-pillars without damaging them technically unfeasible at present. However, to physically address the core question—the influence of phonon scattering intensity on c-axis resistance—we devised a high-temperature substitution experiment.

Experimental Setup: We utilized a commercial MCH ceramic heater (30×30×2 mm) compatible with our high-precision micro-manipulation system. The heater provided a stable surface temperature of 100 ℃ (373 K). We performed four-probe measurements on the three key sample types (HOPG, locked HOPG and ESCG) at Room Temperature (293 K) and High Temperature (373 K), as shown in Figure S12a&S12b.

- Results and Analysis: As shown in Figure S12c, the temperature-dependent behaviors of the three samples exhibit distinct trends that correlate with their stacking order: (i) HOPG, the resistance decreased significantly at higher temperatures (negative temperature correlation). This confirms that in stacks with RM and SF, transport is limited by interfacial energy barriers. Phonon-assisted hopping or tunneling at higher temperatures facilitates carrier transport across these barriers, thereby reducing resistance. (ii) Locked HOPG, the resistance also decreased, but the magnitude of the drop was smaller than in pristine HOPG. This is consistent with the elimination of RM barriers, leaving only SFs to dominate the thermal activation process. (iii) ESCG, in stark contrast, the resistance increased at higher temperatures (positive temperature correlation), albeit slightly. This behavior is characteristic of metallic-like coherent transport, where increased phonon scattering disrupts the coherent electron paths in the perfect AB stacking, leading to higher resistance.

These high-temperature measurements effectively isolate the influence of phonon contributions. They confirm that phonon assists transport in incoherent systems (lowering resistance in HOPG and locked HOPG) while acting as a scattering source in coherent systems (raising resistance in ESCG).

## Supplementary Discussion 3: Low-temperature measurements on bulk samples and consistency with historical studies

To further validate the distinct transport mechanisms inferred from our micro-pillar measurements, we performed low-temperature characterization on bulk graphite samples and cross-referenced our findings with historical literature.

Experimental setup and results: Using a vacuum low-temperature probe station (Semishare SCG-0-4) with liquid nitrogen cooling, we measured a bulk HOPG block (10×10×1 mm) and a bulk ESCG flake (5×5×0.03 mm) using a four-probe configuration (Figure S13a). In this configuration, the bulk graphite samples were attached to an iron sheet with conductive silver paste to connect its lower surface. As shown in Figure S13b, the bulk samples exhibited the expected trends: HOPG showed a negative correlation, while ESCG showed a positive correlation.

COMSOL simulation: Given the vast size difference between the bulk samples and the point-probes, we performed COMSOL finite element simulations to interpret the resistivity. (i) Using a 2D axisymmetric model (Figures S14a& S14b) with anisotropic conductivity ($\sigma_{a}=\sigma_{b}=2E6 S/m$, $\sigma_{c} \mathrm{for} \mathrm{HOPG}=5E2 S/m$ and $\sigma_{c} \mathrm{for} \mathrm{HOPG}=2E4 S/m$), we found that the potential drop is highly localized near the probe tips (Figures S14c&S14d). (ii) To simplify the analysis, we here define two effective measurement geometry of radius and depth for estimation, where the potential drops to 1/10th of its peak, ($R_{\mathrm{eq}}=40 \mu m$, $Z_{\mathrm{eq}}=0.6 \mu m$ for bulk HOPG, and $R_{\mathrm{eq}}=30 \mu m$, $Z_{\mathrm{eq}}=3 \mu m$ for bulk ESCG). (iii) The converted resistivities ($\rho_{c-\mathrm{HOPG}}=0.32 \times{10}^{-3}\Omega\cdot m$, $\rho_{c-\mathrm{ESCG}}=1.2 \times{10}^{-5}\Omega\cdot m$) basically align with our micro-pillar measurements reported in the main text. Thus, the temperature trends observed in bulk data reflect the intrinsic change of the system's c-axis resistance.

Consistency with historical data: In fact, previous researchers have conducted systematic temperature-dependent tests on bulk HOPG and single-crystal graphite. All their results confirm the negative temperature correlation behavior (>80 K) in bulk HOPG (including works of Uher et al.^5^ and Matsubara et al.^6^) and positive temperature correlation in bulk single-crystal graphite (Kish graphite in Tsang et al.’s work^7^ and natural single-crystal graphite in Primak et al.’s work^8^). These robust results collectively point to a single fact: phonon contributions exerts a significant influence on the c-axis transport of graphite at room temperature (and over a wide temperature range), and its effect is diametrically opposed for HOPG versus ESCG.

## Supplementary Discussion 4: The “electrical edge” of graphite micropillar

Previous studies on graphite interfaces have proposed differing interpretations of what constitutes an “edge.” One perspective defines the edge geometrically as the region of lattice overlap between two graphite layers,^9-11^ while another considers the physical edge, which often includes amorphous carbon formed during fabrication.^12-14^ In tribological studies, particularly those investigating the origin of friction at graphite interfaces, the primary energy dissipation has been attributed to the physical edge composed of amorphous carbon.^14^ Notably, zero static friction was observed when this amorphous carbon was removed through etching.^13^

In contrast, studies on electrical transport across twisted graphite interfaces have emphasized the role of the geometrical edge defined by lattice overlap, suggesting that it plays a dominant role in interfacial conductance.^9,15^ However, whether the physical edge can contribute significantly to electrical conduction has remained an open question.

To address this, we employed carbon etching techniques to investigate the nature of the “electrical edge” in our system. As shown in Figure S6, we observed negligible variation in electrical resistance before and after etching the edge regions. This result indicates that the physical edge (amorphous carbon) contributes minimally to electrical conduction in our measurements. Consequently, the effective electrical edge is defined by the geometrical lattice overlap, with a characteristic width of approximately 5 nm.

## Supplementary Discussion 5: Assessing the possible influence of in-plane polycrystallinity

The lateral size of HOPG crystallites usually depends on its grade, ranging from several to tens of micrometers. For example, in ZYH-grade HOPG, the average lateral size is approximately 13 μm, as determined by EBSD characterization in Liu et al. (see Supplementary Materials of Ref.^16^). This value is consistent with previously reported self-retracting graphite pillars with lateral dimensions up to 10 μm. In our study, ZYB-grade HOPG was used, and EBSD measurements by Peng et al.^17^ revealed an average lateral crystallite size exceeding 30 μm within the examined region.

Given the relatively large crystallite size, the probability of rotated configurations persisting after rotational locking is expected to be very low, with only a small fraction of graphite pillars intersecting grain boundaries. Indeed, we observed that a few islands could not achieve rotational locking—most likely because their interfaces contained multi-grain configurations that prevented commensurate contact. These data points had been excluded as outliers from **Figures 3**d&**3**e. Therefore, we believe that the locked graphite pillars analyzed in our study are highly representative of the intrinsic behavior.

## Supplementary Discussion 6: The influence of off-axis alignment on the locked-state resistance

As for the alignment issues, we agree that off-axis misalignment between the top and bottom graphene layers is fundamentally difficult to eliminate. However, we minimized it through precise micromanipulation under an optical microscope (×50). Specifically, one tungsten probe was first used to align the graphite mesa laterally, and the other probe was gently placed on top for controlled incremental rotations. This procedure ensured that the off-axis deviation remained within ~0.3 μm, as demonstrated for a locked HOPG pillar in Figure S15. Because each rotation step was followed by a separate measurement, fine positional corrections could be performed in real time. Besides, the self-retraction of the RM interface can also help the axis alignment.

What is more important is that, the interfacial resistance of the locked state is intrinsically very low. For a graphite pillar with a radius of 3 μm, such a deviation of 0.3 μm corresponds to a ~39.4% reduction in the locked interfacial area. (The larger the pillar, the smaller the relative area loss.) A simple estimation shows that reducing the locked area by 40% would increase the interfacial resistance by ~67% (from 0.002 Ω·μm² to 0.0033 Ω·μm²). Assuming 10 locked interfaces, the overall resistance increase due to off-axis misalignment would be only ~0.0014 Ω—well within the experimental uncertainty.

Therefore, the residual off-axis offset exerts a negligible influence on the final locked-state resistance.

## Supplementary Discussion 7: The stacking structures of ESCG, HOPG, and locked HOPG

The stacking characteristics of both ESCG and HOPG have been extensively investigated in previous studies, and there is now a general consensus on their structural nature:

1. ESCG with perfect AB stacking: The ESCG used in this study originates from the new epitaxial growth process developed by Zhang et al.^18^ in 2022. In that work, atomically resolved scanning transmission electron microscopy (STEM) and selected area electron diffraction (SAED) revealed that “*…the adjacent layers are in a perfect AB-stacking structure*”. This is because material growth follows the principle of minimum energy, as they mentioned in the original text: “*the newly grown graphene layer forms AB stacking with the preformed graphite, as the AB-stacking configuration is the energy minimum state ^19^*”. Subsequently, Ding et al.^20^ further demonstrated that the obtained ESCG possesses “*>99% Bernal (2H) stacking*”: “*the out-of-plane lattice stacking conﬁguration of our ultrapure graphite was conﬁrmed to be Bernal (2H) stacking (with an interlayer spacing of 0.335 nm) through atomically resolved cross-sectional scanning transmission electron microscopy (STEM) measurements at the nanoscale*”. More recently, Zhang et al. ^21^ produced mirror-like large-grain ESCG using an optimized version of the same growth principle, and explicitly stated that their graphite films “*are AB stacked throughout*”. Their structural analyses provided the following key evidence for AB stacking: (i) high-resolution TEM and the corresponding SAED pattern “indicate *the AB stacking of this cross section*”; and (ii) “*the array of spots shown in the Laue pattern of the mirror-like graphite film matches well with the simulated pattern of AB-stacked graphite, indicating that this film is 100% AB stacking*”.

2. HOPG with SFs and RMs: The presence of RMs in HOPG is well established. The seminal observation of microscale structural superlubricity in graphite pillars by Liu et al*.*^16^ relied on the intrinsic RMs within HOPG. Moreover, Wang et al.^22^ directly verified the existence of such RMs by characterizing the relative lattice orientations of the top and bottom graphite surfaces. In our own study, STEM characterizations (**Figure 1**b and Figure S10) also directly reveal rotationally misaligned domains. The existence of SFs in HOPG has likewise been supported by previous works. For instance, Yang et al.^23^ exfoliated graphite films from bulk natural graphite crystals and “*and used Raman spectroscopy to identify the presence of ABC stacking*”, indicative of SFs in the parent graphite. In addition, Koren et al.^24^ demonstrated “*direct experimental observation of stacking fault scattering in highly oriented pyrolytic graphite meso-structures*” through vertical conductivity measurements that the non-Gaussian fluctuations observed in the c-axis resistivity of thin HOPG layers originate from the presence of SFs.

3. Locked HOPG containing only SFs (no RMs): To address this, we conducted cross-sectional STEM characterization of the locked HOPG pillar. The lamella was prepared by focused ion beam (FIB) milling, with its graphite base also inside (Figure S9a). The cross-sectional sample (Figure S9b) includes the Pt cap, the locked graphite layers, and the underlying pristine graphite substrate (schematically shown in Figures S9c&S9d). Interestingly, the locked graphite region exhibits a nearly uniform contrast (Figure S9e), while the buried graphite region shows the characteristic grain contrast of HOPG resulting from twist-angle domain boundaries (Figure S9f). The two dark bands visible in the STEM image originate from minor mechanical cracks induced during FIB preparation, due to the mismatch in thermal expansion coefficients between the Pt cap and graphite, and do not affect our interpretation.

In addition, we also attempted to perform XRD of Laue diffraction measurements on our samples. However, as Laue diffraction is a macroscopic characterization technique with a minimum probing area of ~1 mm, it cannot resolve the structural information of the microscale locked graphite pillar (~5 μm in diameter). Therefore, only bulk ESCG and HOPG samples were characterized. The Laue pattern of ESCG (Figure S17a) exhibits the typical sixfold symmetry, confirming the single-crystalline nature of graphite. This result is consistent with the Laue pattern reported by Liyuan Zhang et al.^21^ for their ESCG sample and their simulated pattern of AB-stacked graphite, further verifying the high-quality AB stacking in our ESCG. The additional fourfold-symmetric spots in the center originate from the underlying silicon substrate used for support during measurement, as confirmed by the control measurement on the bare Si region (Figure S17b). Thus, the influence of unexpected fourfold-symmetric can be safely ignored. For comparison, the Laue diffraction pattern of HOPG (Figure S17c) shows no single-crystal symmetry, consistent with its polycrystalline nature.

## Supplementary Discussion 8: Phonon contributions and their implications for low-temperature transport

Phonon contributions plays a key role in determining the vertical (c-axis) transport in graphite at room temperature, as evidenced by both twist-angle and temperature dependences. (1) Previous studies on twist-angle-dependent interlayer conductance^1,25^ have shown that only at specific commensurate angles, such as 0° and 21.8°, does the interlayer transport remain coherent, exhibiting pronounced conductance peaks^26^. At other twist angles, the conductance decreases gradually with rotation from 0° to 30°, consistent with phonon-assisted incoherent transport^27^. We can imagine that if we do the same experiment at low temperature, we will probably detect that the coherent electrical transport described by the theoretical prediction^26^ is only achieved at discrete common angles, and other angles become non-conducting, which is similar to the variable temperature experiments at several angles in the experimental literature^28^. (2) A contrasting temperature dependence has also been observed in different graphite systems^6^ (see Supplementary Discussions 2&3 for details): in HOPG, the c-axis resistivity decreases with increasing temperature, consistent with phonon-assisted incoherent tunneling; whereas in natural graphite, the resistivity increases with temperature, reflecting phonon scattering that disrupts coherent transport.

Regarding the effect of stacking faults, previous study^24^ suggest that higher temperatures facilitate electron hopping across local energy barriers$\sim exp(U/{k_{B}T})$, leading to a negative temperature dependence of resistance. As for the contribution of edges, it primarily arises from localized edge states, which form metallic conduction paths^9,29^ and thus might display a metallic-like temperature dependence—i.e., resistivity increasing with temperature.

Therefore, at low temperatures—where phonon-assisted processes are largely suppressed—we would expect that (1) the resistivity of single-crystal graphite remains comparable to or slightly lower than that at room temperature, still dominated by coherent transport; and (2) in HOPG containing RM and SF, or in rotationally locked HOPG with SF, the resistivity would increase owing to the loss of phonon-assisted tunneling, with edge contributions becoming more prominent.

**References**

1 Koren, E. et al. Coherent commensurate electronic states at the interface between misoriented graphene layers. *Nature Nanotechnology* **11**, 752-757 (2016).

2 Dienwiebel, M. et al. Superlubricity of Graphite. *Physical Review Letters* **92**, 126101 (2004).

3 Wang, Y. et al. Intrinsic Interlayer Shear Strength of Graphite. *Journal of the Mechanics and Physics of Solids*, 105853 (2024).

4 Yang, F. et al. Ultrahigh Thermal Conductance across Superlubric Interfaces in Twisted Graphite. *Physical Review Letters* **134**, 146302 (2025).

5 Uher, C., Hockey, R. L. & Ben-Jacob, E. Pressure dependence of the c-axis resistivity of graphite. *Physical Review B* **35**, 4483-4488 (1987).

6 Matsubara, K., Sugihara, K. & Tsuzuku, T. Electrical resistance in the c direction of graphite. *Physical Review B* **41**, 969-974 (1990).

7 Tsang, D. Z. & Dresselhaus, M. S. The c-axis electrical conductivity of kish graphite. *Carbon* **14**, 43-46 (1976).

8 Primak, W. & Fuchs, L. H. Electrical Conductivities of Natural Graphite Crystals. *Physical Review* **95**, 22-30 (1954).

9 Oz, A., Dutta, D., Nitzan, A., Hod, O. & Koren, E. Edge State Quantum Interference in Twisted Graphitic Interfaces. *Advanced Science* **9**, 2102261 (2022).

10 Liao, M. et al. UItra-low friction and edge-pinning effect in large-lattice-mismatch van der Waals heterostructures. *Nature Materials* **21**, 47-53 (2022).

11 Li, Y., He, W., He, Q.-C. & Wang, W. Contributions of Edge and Internal Atoms to the Friction of Two-Dimensional Heterojunctions. *Physical Review Letters* **133**, 126202 (2024).

12 Hu, H., Wang, J., Tian, K., Zheng, Q. & Ma, M. The effects of disordered edge and vanishing friction in microscale structural superlubric graphite contact. *Nature Communications* **15**, 10830 (2024).

13 Li, T. et al. Toward Zero Static Friction at the Microscale. *Physical Review Letters* **133**, 236202 (2024).

14 Qu, C. et al. Origin of Friction in Superlubric Graphite Contacts. *Physical Review Letters* **125**, 126102 (2020).

15 Dutta, D., Oz, A., Hod, O. & Koren, E. The scaling laws of edge vs. bulk interlayer conduction in mesoscale twisted graphitic interfaces. *Nature Communications* **11**, 4746 (2020).

16 Liu, Z. et al. Observation of Microscale Superlubricity in Graphite. *Physical Review Letters* **108**, 205503 (2012).

17 Peng, D. et al. Load-induced dynamical transitions at graphene interfaces. *Proceedings of the National Academy of Sciences* **117**, 12618-12623 (2020).

18 Zhang, Z. et al. Continuous epitaxy of single-crystal graphite films by isothermal carbon diffusion through nickel. *Nature Nanotechnology* (2022).

19 Mostaani, E., Drummond, N. D. & Fal’ko, V. I. Quantum Monte Carlo Calculation of the Binding Energy of Bilayer Graphene. *Physical Review Letters* **115**, 115501 (2015).

20 Ding, M. et al. Ultrapure Graphite from Solid Refining. *Advanced Materials* **n/a**, 2500461 (2025).

21 Zhang, L. et al. Synthesis and properties of mirror-like large-grain graphite films. *Nature Communications* **16**, 7180 (2025).

22 Wang, K., Qu, C., Wang, J., Quan, B. & Zheng, Q. Characterization of a Microscale Superlubric Graphite Interface. *Physical Review Letters* **125**, 026101 (2020).

23 Yang, Y. et al. Stacking Order in Graphite Films Controlled by van der Waals Technology. *Nano Letters* **19**, 8526-8532 (2019).

24 Koren, E., Knoll, A. W., Lörtscher, E. & Duerig, U. Direct experimental observation of stacking fault scattering in highly oriented pyrolytic graphite meso-structures. *Nature Communications* **5**, 5837 (2014).

25 Chari, T., Ribeiro-Palau, R., Dean, C. R. & Shepard, K. Resistivity of Rotated Graphite–Graphene Contacts. *Nano Letters* **16**, 4477-4482 (2016).

26 Bistritzer, R. & MacDonald, A. H. Transport between twisted graphene layers. *Physical Review B* **81**, 245412 (2010).

27 Perebeinos, V., Tersoff, J. & Avouris, P. Phonon-Mediated Interlayer Conductance in Twisted Graphene Bilayers. *Physical Review Letters* **109**, 236604 (2012).

28 Kim, Y. et al. Breakdown of the Interlayer Coherence in Twisted Bilayer Graphene. *Physical Review Letters* **110**, 096602 (2013).

29 Wang, Y. et al. Bulk and edge properties of twisted double bilayer graphene. *Nature Physics* **18**, 48-53 (2022).
